# Management of andrological disorders from childhood and adolescence to transition age: guidelines from the Italian Society of Andrology and Sexual Medicine (SIAMS) in collaboration with the Italian Society for Pediatric Endocrinology and Diabetology (SIEDP) – part-2

**DOI:** 10.1007/s40618-026-02849-9

**Published:** 2026-02-28

**Authors:** Marco Bonomi, A. Balsamo, C. Bizzarri, D. Gianfrilli, R. Pivonello, G. Russo, E. Sbardella, G. Corona, A. M. Isidori, S. Cianfarani, Vincenzo Rochira

**Affiliations:** 1 Department of Medical Biotechnology and Translational Medicine, University of Milan, Milan, Italy; 2 Department of Endocrine and Metabolic Diseases, IRCCS Istituto Auxologico Italiano, Via Mosé Bianchi, Milan, 90-20149 Italy; 3 Alma Mater Studiorum, University Hospital S. Orsola Malpighi, Bologna, Italy; 4 Endocrinology and Diabetes Unit, Bambino Gesù Children’s Hospital, IRCCS, Rome, Italy; 5 Department of Experimental Medicine, Sapienza University of Rome, Rome, Italy; 6 Centre for Rare Diseases (Endo-ERN Accredited), Policlinico Umberto I, Rome, Italy; 7 Department of Clinical Medicine and Surgery, University of Naples Federico II, Naples, 80131 Italy; 8 Department of Pediatrics, IRCCS San Raffaele Scientific Institute, Milan, Italy; 9 Endocrinology Unit, Azienda Usl, Bologna, Italy; 10 Department of Systems Medicine, University of Rome ‘Tor Vergata’, Rome, Italy; 11 Endocrinology & Diabetes Unit, IRCCS ‘Bambino Gesù’ Children’s Hospital, Rome, Italy; 12 Department of Women’s and Children’s Health, Karolinska Institutet, Stockholm, Sweden; 13 Endocrinology, Department of Biomedical, Metabolic and Neural Sciences, University of Modena and Reggio Emilia, Modena, Italy; 14 Unit of Endocrinology, Department of Medical Specialties, Azienda Ospedaliero-Universitaria di Modena, Ospedale Civile di Baggiovara, Modena, Italy

**Keywords:** Cryptorchidism, Micropenis, DSD, Testicular Tumors, Hypospadia, Testicular torsion, Orchiepididymitis

## Abstract

**Purpose:**

Andrological pathologies in adulthood often originate from conditions arising during childhood, adolescence, or even as early as the gestational and neonatal periods. Despite their clinical relevance, pediatric andrological disorders remain underrepresented in the literature and no shared position statements or comprehensive guidelines are currently available. The present paper complements a previous publication by the same societies, entitled “*Management of andrological disorders from infancy to adolescence and transitional age: Part-1 – The SIAMS–SIEDP position statement”*, by further expanding on these topics.

**Methods:**

SIAMS, in collaboration with SIEDP, convened a multidisciplinary expert task force to develop updated guidelines on the diagnosis and management of andrological disorders from childhood through adolescence and into the transitional age. The resulting recommendations were formulated using the Grading of Recommendations, Assessment, Development, and Evaluation (GRADE) system.

**Results:**

In this *Part-2* of the guidelines, a comprehensive literature review was conducted, focusing on English-language articles related to “cryptorchidism,” “micropenis,” “hypospadias,” “testicular tumors,” “epididymitis,” “orchitis,” “differences/disorders of sexual development,” and “testicular torsion”. For each condition, three key aspects were analyzed: diagnosis, clinical management, and treatment. Based on this analysis, specific recommendations and suggestions were developed for each disorder.

**Conclusions:**

The multidisciplinary guidelines presented in this paper complement those published in the previous Part-1 document. Developed through collaboration among leading medical societies in the field, this joint effort led to a consensus on a set of practical recommendations and suggestions aimed at supporting healthcare professionals in optimizing both andrological and overall health during the transitional age.

## Introduction

Many of the andrological conditions encountered in adult patients—ranging from genital, hormonal, reproductive, and oncological issues to sexual and psychological aspects—originate during childhood, adolescence, or even as early as the gestational and neonatal periods. For this reason, andrology should not be regarded solely as a discipline of adulthood, but rather as a medical specialty concerned with male health and reproductive function across the entire lifespan, starting from birth—or even from fetal life—and extending into aging.

It is well established that the male gonads, along with hypothalamic gonadotropin releasing hormone (GnRH)-secreting neurons and pituitary gonadotroph cells, are highly sensitive to a wide range of influences—including genetic, environmental, chemical, and physical factors—that may disrupt their proper development and function during critical windows such as gestation, the neonatal period, puberty, and the transitional age [1–3]. In this context, andrological evaluation by a pediatric endocrinologist or an andrologist is recommended throughout the prepubertal, pubertal, and postpubertal stages, particularly when indicated by a neonatologist or primary care pediatrician during routine screening visits. Careful assessment in neonates, children, and adolescents enables early diagnosis of a wide spectrum of andrological disorders, including disorders of sexual development, structural anomalies of the genital tract, normal and abnormal pubertal development, undescended testes, genital tumors, and disturbances of gonadal function, with implications for growth, virilization, and fertility. When not promptly diagnosed and treated, these conditions may result in long-term adverse effects on male reproductive health.

Despite their clinical relevance, the current literature on pediatric and adolescent andrological disorders remains limited and fragmented, often focusing on isolated conditions and overlooking the critical transitional period [4–12]. Moreover, effective collaboration between pediatric endocrinologists and andrologists remains essential to ensure appropriate continuity of care during the transition age. This period, following the completion of pubertal development up to young adulthood, involves the transfer of patients from pediatric to adult healthcare services and requires careful attention to provide continuous care and appropriate management of age-specific health issues. The involvement of andrologists and adult endocrinologist may occur at different ages (even early during childhood) according to organizational setting, which may vary across Countries.

While *Part-1* of these guidelines focused on outlining the general framework and highlighted key issues related to andrological care during development [13], the present *Part-2* expands this work by providing evidence-based recommendations on other specific andrological conditions. This joint effort by the Italian Society of Andrology and Sexual Medicine (SIAMS) and the Italian Society of Pediatric Endocrinology and Diabetology (SIEDP) aims to offer practical guidance for the diagnosis, management, and treatment of a wide range of disorders affecting the male population from childhood through the transitional age. Pediatric care systems at both the primary and secondary levels vary widely across Europe [14, 15]. Therefore, these guidelines, developed by physicians practicing in Italy, are primarily tailored to the Italian context, although they include general recommendations that may also be applicable to other countries and can be adapted to specific local circumstances.

## Materials and methods

A literature search of articles in English for the term “cryptorchidism”, “vanishing testis”, testicular agenesis”, “micropenis”, “hypospadia”, “testicular tumors”, “epididymitis”, “orchitis”, “differences/disorders of sexual development” and “testicular torsion” has been performed. In this guideline, we will provide recommendations regarding the evaluation and management of different andrological disorders during childhood and transitional age based on the GRADE (Grading of Recommendations, Assessment, Development, and Evaluation) system for grading both the quality of evidence and the strength of recommendations [16]. According to this system, the strength of recommendation will be divided into ‘strong’, indicated by the number 1 and ‘we recommend’, and ‘weak’ indicated by the number 2 and ‘we suggest’. The grading of the quality of evidence is denoted as follows: ⊕○○○ for very low-quality evidence; ⊕⊕○○ for low quality; ⊕⊕⊕○ for moderate quality; and ⊕⊕⊕⊕ for high quality [16].

### Minipuberty

This paragraph aims to recall some key concepts related to the critical phase of minipuberty, which are further addressed in the subsequent subsections of these guidelines. Minipuberty is a physiological phase that occurs in the first few months of life, during which the hypothalamic-pituitary-gonadal (HPG) axis, active during fetal development but suppressed at birth, reactivates for a brief period. This leads to measurable increases in gonadotropins, such as luteinizing hormone (LH) and follicle-stimulating hormone (FSH), as well as gonadal steroids like testosterone (T) in male infants and estradiol (E2) in female infants [17–19]. Minipuberty typically begins in the first few weeks of life, peaking between 4 and 12 weeks of age. In males, the T surge gradually diminishes by 4 to 6 months, whereas in females, E2 circultating levels may persist for a longer duration. During this period in males, LH stimulates Leydig cells in the testes to produce T peaking in the 2nd − 3rd month of life when T values may reach those of fertile adult men. T is crucial for penile and scrotal growth, testicular descent, and early germ cell maturation. FSH stimulates Sertoli cells, increasing inhibin B and promoting spermatogonial development and testicular growth.

In females, FSH levels are higher than in males during minipuberty, stimulating early ovarian follicular activity. Ovarian granulosa cell products inhibin B and anti Mullerian hormone (AMH) transiently increase at the time of the postnatal gonadotrophin surge. Unlike T levels in boys that show a clear peak at 1–3 months of age and then decrease steadily towards the age of 6 months, E2 levels in girls fluctuate, probably reflecting cyclic maturation and atrophy of ovarian follicles. E2 levels decrease during the 2nd year of life. Early postnatal E2 secretion contributes to the development of the uterus and breast tissue, although its clinical manifestations remain subtle.

Minipuberty is an essential diagnostic window for evaluating the functional integrity of the HPG axis in conditions like Congenital Hypogonadotropic Hypogonadism (CHH) and Disorders of Sex Development (DSDs) [18, 20, 21]. Hormonal testing during this phase, such as measuring serum LH, FSH, T, Inhibin B, and AMH, provides valuable insights into gonadal function without the need for stimulation tests. In male infants with cryptorchidism or micropenis, evaluating minipuberty can help in the detection of underlying HPG axis abnormalities and guide early interventions. In cases of CHH, the absence of the gonadotropin and sex steroid surge is a hallmark feature [18]. Disorders of androgen receptor sensitivity or enzymatic defects in T synthesis may also manifest during this phase (normal/elevated testosterone levels associated with relatively elevated gonadotropins in androgen receptor defects, reduced T levels with variable gonadotropins in enzymatic defects of gonadal steroidogenesis).

In cases where androgen deficiency is detected during minipuberty, early treatment with low-dose T may be considered to support normal genital development [17–19]. There are also more limited reports suggesting the use of gonadotropin therapy to replicate the missed minipuberty [20, 22]. Nonetheless, it should be emphasized that both mentioned therapeutic approaches are currently considered off label. For female infants, hormonal abnormalities during this time could signal potential reproductive challenges, warranting close follow-up [18].

## Cryptorchidism

### Definition and epidemiology

Cryptorchidism (or undescended testis) is defined as failure of one or both testicles to complete their descent into the lower part of the scrotum (Table 1) [23]; it is the most common genital disorder identified at birth [24, 25].

**Table 1:** Cryptorchidism: definitions

Cryptorchidism (undescended testis)	Failure of one or both testicles to complete their descent into the lower part of the scrotum; the testicles are located along the normal testicular descent pathway (intra-abdominal, inguinal, supra-scrotal or high scrotal position)
Congenital anorchia (vanishing testis syndrome)	The absence of testicular tissue in 46,XY phenotypic males
Acquired cryptorchidism (ascending testis)	One or both testicles cannot be returned into the scrotum after previously having been localized in a stable scrotal position
Ectopic testis	Testicle outside its normal route, in a perineal, femoral, or pubo-penile position, or at a crossed scrotal position (unilateral location of both testes).
Retractile testis (hypermobile testes)	Testis located in the upper scrotum or lower inguinal canal that can be moved by manual reduction into the scrotum, where it remains until stimulation by the cremasteric reflex occurs

Prevalence of cryptorchidism at birth has been estimated between 1.8 and 8.4% among at term and appropriate for gestational age (AGA) babies [23]. Given the relatively late migration of testes through the inguinal canal into the scrotum, prevalence in preterm and/or small for gestational age (SGA) newborns may reach 45%, as inversely related to gestational age [24]. Prevalence at the age of 3 months and 1 year drops to 0.9–1.6% and 1.0–1.5%, respectively, due to the spontaneous testicular descent in around 40% of infants, usually within the first 3 months of age [23–26]. Congenital cryptorchidism is generally diagnosed at birth, but a few children with cryptorchidism may be referred late, either because of missed diagnosis or because they have an ascending testicle (acquired cryptorchidism) [24]. Acquired cryptorchidism is the condition in which one or both testicles cannot be returned into the scrotum after previously having been localized in a stable scrotal position (Table 1) [27]. Its prevalence is 0.6%–7% between 18 and 36 months of age and 1.1%–2.2% between 6 and 13 years, with a peak around 8 years of age [24, 28, 29].

Cryptorchidism should be differentiated from congenital anorchia or vanishing testis syndrome (Table 1), defined as the absence of testicular tissue in 46,XY phenotypic males [30]. The condition is unilateral in 97% of cases and accounts for about 10% of cases in which the testis is absent from the scrotum or inguinal canal [31]. Müllerian structures are absent and Wolffian structures are normal, but the ipsilateral vas deferens is often rudimentary and the epididymis is absent [32].

The frequency of bilateral anorchia is reported to be 1 in 20,000 phenotypic males [33]. Since testicular function is essential to stimulate the development of the external genitalia, testes must have been normal in the first trimester of gestation. Antenatal or perinatal vascular thrombosis or testicular torsion occurring during later testicular descent into the scrotum have been suggested [34, 35].

Boys with bilateral anorchia are distinguished from those with bilateral abdominal cryptorchidism by imaging (ultrasound and/or magnetic resonance), laparoscopy or surgical exploration, low serum levels of AMH and inhibin B and undetectable T levels after human chorionic gonadotropic (hCG) administration.

Rarely, the testis may be found outside its normal route in a perineal, femoral, or pubo-penile position (ectopic testis), or at a crossed scrotal position (unilateral location of both testes) [36].

Undescended testis should be always distinguished from retractile one, defined as a testicle located in the upper scrotum or lower inguinal canal that can be moved by manual reduction into the scrotum, where it remains until stimulation by the cremasteric reflex occurs (Table 1) [27].

### Pathophysiology

Cryptorchidism is usually an isolated condition of unknown cause, though it can also occur in genetic syndromes (syndromic cryptorchidism). The presence of micropenis and/or hypospadias may indicate impaired secretion or action of hormones critical for genital development and testicular descent [37, 38]. Testicular descent occurs in two phases. The first, transabdominal phase takes place between 8 and 15 weeks of gestation [39]. During this phase, the testis is anchored in the internal inguinal ring by enlargement of the gubernaculum, preventing upward movement as the embryo grows [40]. Regression of the cranial suspensory ligament, which is androgen-dependent, also contributes to gonadal positioning [41].

Animal studies suggest that gubernaculum development is driven by INSL3 and its receptor [41, 42]. however, mutations in these genes are rarely observed in humans [43], reflecting the infrequent disruption of the transabdominal phase [44–46].

The second, inguinoscrotal phase involves the testis migrating from the internal inguinal area to the scrotum [39]. Widening of the inguinal canal, along with gubernaculum shrinkage and intra-abdominal pressure during the seventh month of gestation, guides the testis into the scrotum [35, 47].

This phase is androgen-dependent and may be impaired in androgen receptor defects [48–50] or inborn error of steroidogenesis [23], although causative mutations are rarely identified in isolated cases [51–53].

Although familial clustering has been reported, cryptorchidism is likely polygenic, with no single causative gene identified [54, 55].

Other mechanisms of under-masculinization may also affect testicular descent. In persistent Müllerian duct syndrome, defects of AMH or its receptor can result in abdominal or inguinal testes, confirming AMH’s role in the transabdominal phase [56].

Congenital hypogonadotropic hypogonadism is another common association [57, 58]. Here, hCG can partially compensate for missing LH in utero, explaining why not all affected boys are cryptorchid at birth, while postnatal pituitary gonadotropins may influence testicular position, as seen in secondary (acquired) cryptorchidism [59].

### Clinical picture

A testicle is defined as undescended if it cannot be manipulated, tension-free, into the scrotum at any age. Palpable undescended testes lie below the internal inguinal ring and may be detected along the normal descent pathway in inguinal, supra-scrotal, or high scrotal positions. By contrast, a retractile testis can be manually brought into the scrotum without tension, though it may return to its previous position afterward [60].

About 70% of undescended testes are palpable with experienced examination [61, 62]. The remaining 30% are non-palpable and may reside in inguinal, abdominal, or ectopic locations, while a small fraction cannot be located even after laparoscopic exploration, representing vanishing testes [60].

### Diagnostic evaluation

#### Recommendations and Suggestions

**R1.1** We recommend always assessing testicular position at birth in all newborns and at 3 and 6 months of age in those with congenital cryptorchidism, for the known possibility of spontaneous descent within the first 6 months of life (1, ⊕⊕⊕⊕).

**R1.2** We recommend that primary care pediatricians and/or general practitioner should palpate testes for position at each recommended well-child visit (1, ⊕⊕⊕○).

**R1.3** We recommend a prompt referral to a tertiary care center for all male newborns with bilateral non-palpable testes, especially when associated with other hallmarks of disorders of sexual development (i.e. hypospadias, microphallus, bifid scrotum) (1, ⊕⊕⊕○).

#### Evidence

The diagnostic work-up of isolated monolateral cryptorchidism is still predominantly clinical. A thorough physical exam should be sufficient to diagnose undescended testes [63].

In the hands of an experienced clinician, more than 70% of cryptorchid testes are palpable by physical examination. In the remaining 30% of cases the gonads are non-palpable and the challenge is to confirm absence or presence of the gonads and their characteristics [64].

Acquired cryptorchidism is easily distinguished from congenital cryptorchidism if scrotal testicular position has been documented since birth. Retractile testes have been described as more at risk for secondary ascent [65, 66]. Acquired cryptorchidism is more common in boys with a history of proximal hypospadias suggesting a common mechanism related to aberrant androgen signaling [65]. Acquired cryptorchidism may also represent a complication of inguinal hernia surgical correction [67].

Ultrasound shows limited sensitivity and specificity in localizing non-palpable testes, with 45% sensitivity and 78% specificity [68]. Intra-abdominal testes have been localized at surgical exploration in 49% of boys with a non-palpable testicle and a negative ultrasound [68]. The use of computed tomography is limited due to cost and radiation exposure. Magnetic resonance imaging demonstrates better sensitivity and specificity, but its use is discouraged due to the high cost, limited availability, and the requirement for anesthesia [69, 70].

Unilateral or bilateral cryptorchidism associated with micropenis, suggest congenital hypogonadotropic hypogonadism, isolated or in the context of a multiple pituitary hormone defect [71].

Unilateral or bilateral cryptorchidism associated with hypospadias and/or bifid scrotum are suggestive of DSD [72]. A newborn with male phallus or hypospadias, atypical external genitalia and bilateral non-palpable gonads is potentially a genetic female (46,XX) with congenital adrenal hyperplasia due to 21-hydroxylase deficiency or another rare defect of adrenal and gonadal steroidogenesis (11 beta-hydroxylase deficiency, steroidogenic acute regulatory protein (StAR) or cholesterol side-chain cleavage enzyme defect). These newborns are at risk for life threatening salt wasting crises during the first weeks of life and need urgent hormonal and genetic assessment (at least karyotype analysis), followed by further genetic analyses to define the specific steroidogenesis defect and its treatment [73].

The assessment of karyotype and gonadal function is strongly recommended in the definition of complex cases, especially when cryptorchidism is bilateral and/or part of a complex DSD [74, 75].

Infant boys with 46,XY karyotype, bilateral non-palpable testes, normal penile development or micropenis without hypospadias need extensive evaluation to differentiate vanishing testis syndrome (bilateral congenital anorchia) from bilateral abdominal testes [76].

#### Remarks

Clinical examination should be always performed with the child placed in a supine and frog-legged position on the examination bed, or on the parent’s lap [61, 62]. The clinician should examine the baby in a warm room with warm hands, standing to the patient’s right side. The left hand is initially placed lateral to the deep inguinal ring and moves along the inguinal canal up to the pubic tubercle, exerting a moderate pressure to push the testicle down. This maneuver overcomes the cremasteric reflex, which naturally pulls the testicle away from the scrotum. When the left hand has reached the pubic tubercle, the right hand can locate the testis and gently pull it down to the base of the scrotum. The same process can be repeated to locate the testis on the opposite side [60].

Imaging studies do not significantly help diagnosis and rarely assist in decision-making. Diagnostic laparoscopy (or open surgical exploration) should be performed on all non-palpable unilateral and most bilateral cryptorchid patients [77].

Corrected age should be always used to determine optimal surgical timing in infants born preterm (< 32 gestational weeks) (*corrected age = chronological age (weeks) minus the number of weeks the infant was born before the due date*).

A basal blood sampling (for LH, FSH, testosterone, AMH, inhibin B) performed between 4 and 16 weeks of life (minipuberty) can assess gonadal function and help the diagnosis of hypogonadism or DSD, with greater accuracy than stimulation tests performed later in childhood. During minipuberty, infants with anorchia obviously do not have any postnatal surge of T, AMH and inhibin B are undetectable, while FSH and LH are elevated [18, 71]. On the contrary, isolated congenital hypogonadotropic hypogonadism is suggested in cryptorchid infants with micropenis and low or undetectable levels of gonadotrophins and T [18, 71, 78, 79].

After minipuberty, the hypothalamic-pituitary-gonadal axis remains quiescent until true puberty [18, 71, 78, 79]. In the past decades, stimulation tests with hCG were recommended to assess Leydig cell function in children with bilateral non-palpable testes outside minipuberty [80]. Nevertheless, the failure of T to increase after hCG stimulation is not diagnostic of anorchia, because undescended dysgenetic testes may not respond to hCG stimulation [81]. On the contrary, undetectable levels of inhibin B and AMH associated with elevated gonadotropin levels are suggestive of isolated anorchia in a phenotypic 46,XY male with bilateral non-palpable testes [74, 81].

### Therapeutic management

#### Recommendations and suggestions

**R1.4** Children with persistently undescended testis after 6 months of age should be referred to surgical evaluation, the best surgical timing being between 6 and 12 months of age to improve potential for fertility and reduce the risk for testicular cancer (1, ⊕⊕⊕○).

**R1.5** We recommend annual follow-up until puberty to monitor for a possible secondary ascent in boys with retractile testes, avoiding any medical treatment (1, ⊕⊕⊕○).

**R1.6** We suggest considering hormonal therapy only in infants with congenital hypogonadotropic hypogonadism to induce minipuberty, improve surgical outcome, and sometimes induce testicular descent (2, ⊕○○○).

**R1.7** Individuals with a history of cryptorchidism and delayed surgical correction (> 12–18 months) should be informed by clinicians about potential long-term risk of infertility (in bilateral cryptorchidism) and testicular cancer to ensure long-term andrological follow-up (clinical principle). (Expert opinion).

#### Evidence

Orchidopexy is the only successful therapy to relocate the testis into the scrotum [77, 82].

Spontaneous descent of the testes into the scrotum during the first months of life highlights the need to repeat clinical examination at 3 and 6 months of age before considering surgical correction [23–26, 83].

Patient age at orchiopexy is directly related to the severity of germ cell and Leydig cell depletion [84, 85]. Non-palpable testes carry the highest risk of germ cell depletion [86]. Adverse histologic features (e.g. loss of germ cells) are similar in congenital cryptorchidism and acquired cryptorchidism [87]. Early surgery improves testicular volume, but the benefits on future fertility are still uncertain [37]. Late complications of surgery include testicular atrophy (up to 15% for intra-abdominal testis) and testicular re-ascent requiring revision surgery (10% for intra-abdominal testis) [88].

Early data suggested a significantly higher risk of carcinoma in abdominal testes [89]. The relative risk of developing testicular cancer in individuals with isolated cryptorchidism has been estimated to be 2.90, even after early surgical correction [90]. Nonetheless, is also knonw that this risk is generally lower in congenital forms of hypogonadism (such as congenital hypogonadotropic hypogonadism) [91].

Hormonal therapy with hCG or LHRH in cryptorchidism is controversial and evidence is lacking on any clear benefit in improving fertility [77, 92, 93]. Available data on the efficacy of hormonal therapy to induce testicular descent (FSH + hCG/LH, GnRH) have demonstrated an inadequate efficacy and a high risk of recurrence [37, 94, 95]. Around 60% of men with congenital central hypogonadism (lacking minipuberty) achieve adult testicular volume and semen quality with gonadotropin replacement therapy [96, 97]. A proposed approach to enhance the fertility potential of these patients is to mimic the hormonal milieu of minipuberty, with the aim of promoting Sertoli and germ cell proliferation and initial differentiation [18, 71, 96].

Short-term combined gonadotrophin therapy stimulates normal levels of T, inhibin B and AMH in infants with congenital hypogonadotropic hypogonadism [71, 98, 99]. In these children, gonadotrophin treatment might also facilitate the surgical approach to undescended testes and contribute to reduce surgical complications by the increase of testicular volume [18, 99].

#### Remarks

Hormonal therapy of cryptorchidism should be considered only in cryptorchid infants with congenital hypogonadotropic hypogonadism, with the aim of mimicking minipuberty, even if there is not yet consensus to use this approach as a routine therapy.

Short-term gonadotropin therapy in these infants aims to improve future testicular development and fertility potential, but it also facilitates surgical approach and outcome. A practical approach with daily injections of recombinant LH and rFSH, has been proposed. Treatment should be started before age 6 to 9 months, at an initial dose of 37.5 IU rLH and 75 IU rFSH per day. This dose can be increased 1 or 2 weeks later, if serum LH concentrations do not reach the physiological range [18]. Data on efficacy and safety of with hCG area also available [100, 101].

Successful scrotal repositioning of the testis may reduce, but not completely prevent, long-term issues of infertility and testicular cancer [102].

A long-term andrological follow-up addressing fertility issues and long-term risk of testicular cancer is appropriate for patients with a history of cryptorchidism, especially if surgical correction was performed late and/or cryptorchidism was bilateral. Diagnostic work-up should include periodical clinical evaluation, hormonal assessment, and imaging studies [103–105].

## Disorders of sex development (DSD)

The aim of this chapter is to provide a general approach to a child with a DSD. Due to the high heterogeneity of etiologies of DSDs together with the rapid evolution of knowledge, management and even terminology in this field, we suggest referring also to available international guidelines or position statements for more insights into diagnostic workup and treatment of these conditions [106–115].

### Epidemiology

The term “DSD” defines a large and heterogeneous group of congenital conditions in which development of chromosomal, gonadal, or anatomic sex is atypical.

This nomenclature and the subsequent classification of distinct DSDs was introduced in 2005, following the Chicago consensus conference [116]. Nomenclature remains controversial and now other terms are also used such as “differences” or “variations of sex development”; these terms may help to introduce the concept of the range of variation that may occur in sex development, as underlined by some advocacy groups and some patients [106, 107]. The current medical classification is largely based on the genetic status of the patient (Table 2) [106, 116].

**Table 2:** Current classification of disorders of sex development (DSD) (from [116])

Sex chromosomal DSD	46,XY DSD	46,XX DSD
45,X Turner syndrome and variants	*Disorders of gonadal Development*	*Disorders of gonadal Development*
	*• *Complete or partial gonadal dysgenesis, monogenic forms (for example, SRY, NR5A1 and WT1)	*• *(Ovo)testicular DSD
	*• *Testes regression	*• *Monogenic forms of primary ovarian insufficiency (mutations in genes involved in gonadal (ovarian) development; for example NR5A1 and WT1)
	*• *Ovotesticular DSD	*• *Syndromic forms
	• Syndromic forms	
47,XXY Klinefelter syndrome and variants	*Disorders of androgen synthesis*	*Disorders of androgen excess*
	*• *Associated solely with androgen biosynthesis defects (mutations or deficiencies in HSD17B3 and SRD5A2, for example)	*• *Aromatase (CYP19A1) deficiency
	*• *Associated with congenital adrenal hyperplasia and early androgen biosynthesis defects (mutations and/or deficiencies in STAR, CYP11A1, HSD3B2, POR and CYP17A1)	*• *Congenital adrenal hyperplasia (mutations and/or deficiencies in CYP21A2, HSD3B2, CYP11B1 and POR)
	*• *Associated with placental insufficiency or endocrine disruption	• Luteoma
	*• *Syndromic forms (for example, Smith–Lemli–Opitz)	*• *Latrogenic
45,X/46,XY Mixed gonadal dysgenesis	*Disorders of androgen Action*	*Unclassified disorders*
	Complete and partial androgen insensitivity	• MRKH type I and II syndrome• Complex syndromic disorders
46,XX/46,XY Chimerism	*Persistent Müllerian duct syndrome* Due to mutations or deficiencies in *AMH* and AMHR*2*	
	*Unclassified disorders*	
	*•* Hypospadias of unknown origin	
	*•* Epispadias	
	*• *Complex syndromic disorders	

The birth prevalence of genital atipicity may be as high as 1 in 300 births, but the birth prevalence of a condition that may lead to difficulties in gender attribution and require expert examination may be as low as 1 in 5000 births [117]. The age at clinical presentation of a patient with a DSD is mostly at birth, but can be diagnosed also in infancy, late adolescence or even adulthood [110, 118]. Sometimes a DSD can be already suspected in the prenatal age [108].

### Pathophysiology

The term “sex development” refers to the biological processes leading to the morphological and functional acquisition of sexual characteristics. It is driven by complex, dose- and time-dependent genetic networks that diverge between the sexes from the 7th week of embryonic development and culminate at puberty with reproductive maturity [119, 120].

Dimorphic sexual development involves two sequential processes [121]:

1. “sexual determination” - the differentiation of the undifferentiated gonad into a testis or ovary according to chromosomal sex.
2. “sexual differentiation” - the development of internal and external genitalia and the sex-specific changes occurring at puberty, guided by gonadal hormones.

Gonads originate from the genital ridges, which appear undifferentiated in both sexes around the 4th week of gestation. Sex determination is orchestrated by gene networks, including SRY on the Y chromosome, which activates SOX9 to promote testicular development [122]. Altered timing or dosage of these genes can impair testis or ovary formation [123]. Internal genitalia arise from two parallel ducts: the Wolff (mesonephric) and Müller (paramesonephric) ducts. In the developing testis, testosterone production begins around the 7th–8th week and acts via androgen receptors to form the epididymis, vas deferens, and seminal vesicles. Sertoli cells produce anti-Müllerian hormone (AMH) from the 7th week onward, inducing regression of Müllerian ducts [124]. In the absence of testosterone, Wolff ducts regress; without AMH, Müllerian ducts persist, forming female internal genitalia. External genitalia develop from the cloaca. In males, the genital tubercle forms the penis, the urogenital folds fuse to create the penile urethra, and the genital swellings merge into the scrotum. Testicular descent, partially driven by Leydig cell–derived INSL3, occurs in the last trimester. Dihydrotestosterone (DHT), produced from testosterone by 5α-reductase, directs development of the penis, scrotum, and prostate [125]. While largely androgen-dependent, external genital development also involves other hormones and hormone-independent mechanisms; in the absence of DHT, female-typical external genitalia develop.

### Clinical picture

Several signs characterize the clinical picture of DSDs and are all included among the following criteria used for raising the clinical suspicion of these conditions according to patient’s age (Table 3).

**Table 3:** Age-specific clinical features suggestive of Disorders of Sex Development (DSD)

Newborn	Child and young adult
1. Overt atypical genitalia	1. Previously unrecognized atypical genitalia
2. Apparent female genitalia with an enlarged clitoris, posterior labial fusion, or an inguinal/labial mass	2. Inguinal hernia in a female
3. Apparent male genitalia with bilateral undescended testes	3. Delayed or incomplete puberty
4. Micropenis	4. Virilization in a female
5. Isolated perineal hypospadias	5. Primary amenorrhea, with or without breast development
6. Mild hypospadias with undescended testis	6. Breast development in a male
7. A family history of DSD	7. Gross and occasionally cyclic hematuria in a male
8. A discordance between genital appearance and a prenatal karyotype	8. Infertility

### Diagnostic evaluation

#### Recommendations and suggestions

**R2.1** We recommend a careful and thorough evaluation of the genitals in all newborns at birth aiming to identify atypical genitalia. (1, ⊕⊕⊕⊕).

**R2.2** We recommend that a newborn/child/adolescent with a suspected DSD is referred quickly to a Pediatric Endocrinology tertiary center with a multidisciplinary team experienced in DSDs for a complete care and a comprehensive diagnostic work up (1, ⊕⊕⊕⊕).

**R2.3** We recommend performing genetic testing in all DSD patients (1, ⊕⊕⊕⊕).

**R2.4** We recommend that the family and/or the patient should be promptly offered psychosocial support by a professional with experience in DSD. (1, ⊕⊕⊕⊕).

**R2.5** We recommend that the diagnosis as well as pros and cons of the various intervention options should be communicated to the parents and the patient in a plain and structured communication strategy by the multidisciplinary team. (1, ⊕⊕⊕○).

**R2.6** We suggest the early involvement of support groups. (2, ⊕⊕○○).

#### Evidence

When DSD is suspected at birth or at any age diagnostic procedures should be performed by a multidisciplinary team at a designated excellence center with efficient communication between all its professional areas. An excellence center should have members of all involved areas of expertise inside (pediatric/adult endocrinologists, psychologists, ethicists, pediatric surgeons/urologists, gynecologists, andrologist, geneticists, pathologists, radiologists, laboratorist, sex therapists, and social workers) or alternatively foster a close connection to those disciplines not available at the center itself [106, 110, 112]. Representatives of the different disciplines and specialties should be experienced in the field of DSDs. The center should ensure DSD patient care throughout his (or her) life and ensure the effective transition of care from childhood through adolescence to adulthood [126, 127].

A comprehensive diagnostic evaluation is required to determine the clinical outcome regardless of an immediate indication for treatment [106, 110]. History should be aimed at investigating if there are other family members with atypical genital development or if any unexplained deaths have occurred in the family. Probing for consanguinity may be helpful especially when an autosomal recessive disorder is being considered in the differential diagnosis. A detailed family history regarding fertility, as well as the use of androgenic or estrogenic substances during pregnancy, maternal virilization during pregnancy, and information about procreation may be helpful.

A detailed physical exam is mandatory. In newborn and infants, description of external genitalia is facilitated using specifically designed, quantitative scoring systems. A non-binary version applicable in both boys and girls, has been validated in a European multicentre study (External Genital Score, EGS; Table 4) [128]. The anogenital distance (AGD) correlates with prenatal androgen exposure [129].

**Table 4:** External Genitalia Score (EGS) [128]

EGS	Labioscrotal fusion	Genital tubercle length (mm)	Urethral meatus	Right gonad	Left gonad
3	Fused	> 31	Top of the GT		
2.5		26–30	Coronal glandular		
2			Along the GT		
1.5	Posterior fusion	21–25	At the GT base	Labioscrotal	Labioscrotal
1		10–20	Labioscrotal	Inguino-Labioscrotal	Inguino-Labioscrotal
0.5				Inguinal	Inguinal
0	Unfused	< 10	Perineal	Impalpable	Impalpable

Hormonal diagnostics are essential [130]. Basic values to be determined include adrenal (17-hydroxyprogesterone, cortisol, androstenedione, serum electrolytes) and gonadal function tests (E2, T, DHT, gonadotropins, AMH). The analysis of these hormones should be performed at a specialized laboratory. Currently, chromatographic, mass spectrometric methods are recommended for exact steroid hormone measurements [106, 130, 131]. However, these methods are not yet widely available. Measurement of AMH plays a pivotal role in determining the type and quality of gonadal tissue [132]. In undervirilized 46,XY individuals, low AMH levels suggest gonadal dysgenesis, while normal or high levels indicate androgen insensitivity or synthesis defects. In 46,XX individuals, elevated AMH levels may point to the presence of testicular tissue, as seen in ovotesticular DSD.

Gonadal tests may be repeated during minipuberty in the suspicion of hypogonadism [133]. Additional investigations include hCG and adrenocorticotropin (ACTH) stimulation tests [110]. A complete examination by ultrasound of the pelvic structures as well as the perineal region and of the entire urinary tract is recommended. Magnetic resonance imaging should be reserved for cases when ultrasonography is not informative, when there are abnormalities of the urinary tract, in case of intra-abdominal gonads. For identification of intra-abdominal gonads, laparoscopy may refine the diagnosis. Advantages and risks should be considered before performing a laparoscopy for identification of the gonads and inner genitalia. X-ray based fluoroscopic genitography can be unnecessary. An invasive procedure such as urethrocystoscopy or an inspection of the vagina under anesthesia might be necessary to identify details of the anatomy [134, 135].

Chromosome analysis (including karyotype with fluorescence in-situ hybridization, FISH and array-comparative genomic hybridization, aCGH) is the first step of genetic analyses [136, 137]. The next step includes the use of next-generation sequencing (NGS) assays, designed to sequence multiple DSD genes on a targeted panel in one analysis or whole-exome sequencing (WES) with predetermined filters that target DSD genes [138, 139]. Written consent is mandatory before conducting any procedure. A genetic consultation should outline the possibilities, limits, and problems of such tests.

The psychosocial counseling for parents and patients should be provided for the whole life since it is crucial to promote a positive adaptation to the clinical condition [140–142]. Family counselling to parents and/or patient is a key step in this process and should be integrated at any medical visit [143]. Any aspect of the management should be communicated in a balanced and respectful way by providing risks and benefits of any future choice and avoiding time pressure. Counseling and attendance of children/adolescents with DSD is based on the principle of allowing them to accept their bodies and achieve the best quality of life possible. The child should be informed gradually and repeatedly while it is growing up. Communication must be documented, and a copy of the information should be handed out to the parents. The inclusion of trained peer support in the multidisciplinary DSD team is advisable to the supportive management of patients with DSDs [144, 145]. This inclusion will strengthen the acceptance of DSD and facilitate the sharing of experiences, thereby reducing the stress and isolation felt by patients and their families.

#### Remarks

The management of a newborn, child, or adolescent with a DSD can be complex and often presents a clinical challenge [106, 146, 147]. Initial interactions and communications can have a lasting impact on the patient’s medical journey. Therefore, it is crucial that all healthcare professionals who may encounter such cases possess a thorough understanding of DSDs. Equally important is the management of these cases in specialized reference centers staffed by experienced and competent personnel [106]. When immediate referral is not feasible—for instance, in premature infants requiring neonatal intensive care—it is essential for the attending neonatologist or healthcare provider to promptly consult with specialists at a reference center to coordinate care.

A primary concern in the initial evaluation is the identification or exclusion of adrenal insufficiency, as it represents the only clinical scenario necessitating immediate, life-saving hormone replacement therapy [147]. Congenital adrenal hyperplasia (CAH) due to 21-hydroxylase deficiency (21-OHD) is the most prevalent cause of 46, XX DSD. Diagnosis involves measuring serum 17-hydroxyprogesterone levels, performing karyotyping, and conducting ultrasound examinations by experienced operators. Prompt diagnosis allows for the initiation of critical hormone replacement therapy.

However, 21-OHD CAH is not the sole cause of adrenal insufficiency associated with DSD. Other aetiologies include 11β-hydroxylase deficiency, 3β-hydroxysteroid dehydrogenase deficiency, and P450 oxidoreductase deficiency, which can present in both 46, XX and 46, XY individuals. In 46, XY patients, conditions such as StAR defects, P450scc, and P450c17 deficiencies can lead to DSD presentations [148].

Assessing gonadal function at birth is important, though commonly used laboratory techniques like immunometric assays have limitations, especially in neonates. The adoption of chromatography coupled with mass spectrometry is expected to improve diagnostic accuracy. Re-evaluation during the “minipuberty” period is recommended.

Genetic testing has advanced significantly and is increasingly becoming a first-line diagnostic tool in certain clinical scenarios. Early and accurate genetic diagnosis facilitates targeted management strategies.

In Italy, birth registration, including the assignment of name and sex, must occur within 10 days. In cases of neonates with atypical genitalia, this timeframe may be insufficient to gather comprehensive clinical information for accurate sex assignment. While many aspects of long-term outcomes in individuals with DSD remain under investigation, current data support specific recommendations for sex assignment. For example, in individuals with a 46,XY karyotype and 5α-reductase deficiency or 17β-hydroxysteroid dehydrogenase deficiency diagnosed at birth, current guidelines support assigning sex in accordance with chromosomal and gonadal sex, as is also recommended for individuals with 46,XX CAH. A medically justified and documented delay in birth registration is generally accepted.

Psychological support is an integral component of comprehensive DSD management and should be provided by professionals with expertise in the field. Studies highlight the importance of accessible mental health services throughout the lifespan of individuals with DSD. Multidisciplinary teams should include mental health professionals to address the psychosocial needs of patients and their families.

### Therapeutic management

#### Recommendations and suggestions

**R2.7** We recommend that DSD counseling/therapy should be an interdisciplinary task, performed in centers of excellence aiming to achieve the best quality of life and acceptance of individual anatomy. (1, ⊕⊕⊕O).

**R2.8** We recommend starting immediately replacement therapy when adrenal insufficiency is diagnosed (1, ⊕⊕⊕⊕).

**R2.9** We recommend starting sex hormone replacement therapy in case of documented deficiency at pubertal age or later at the time of diagnosis independently from the underlying cause. (1, ⊕⊕⊕⊕).

**R2.10** We suggest considering GnRH-analogue to block the HPG axis when the subjects start to enter puberty and develops a phenotype that is discordant with the existing gender role, to be started only after in-depth psychological assessment and support. (2 ,⊕○○○).

**R2.11** We suggest that surgical interventions at early age is indicated only when there is a documented risk for the patient’s health and well-being in case of no intervention. (2, ⊕⊕○○).

**R2.12** We recommend that, if surgical procedures are planned, they should be performed by qualified surgeons with proven experience in this field. (1, ⊕⊕⊕○).

**R2.13** We suggest that a long-term follow up should be offered to people who have undergone surgery. (2, ⊕⊕○○).

**R2.14** We suggest a continuous follow-up and screening of both function and oncological risk of the gonads (2, ⊕⊕○○).

**R2.15** We suggest that transitional care should be provided by a multidisciplinary team which should ideally include both pediatric and adult professionals with experience in DSD management. (2, ⊕○○○).

#### Evidence

The therapeutic management of a patient with DSD should be carried out by multidisciplinary teams in centers of excellence. The aim is of achieving the best quality of life and acceptance of individual anatomy and the optimal long-term well-being [106, 107, 110, 118]. However, the therapeutic management is extremely challenging, due to the diagnostic and ethical issues presented by many patients and the difficulty in providing a precise prognosis for any individual patient. These issues must be kept in mind in any decision-making process and in communication with the patients and families. Families and patients, when possible, fully informed, must be directly involved in decision-making process [149].

Adrenal insufficiency is a possible and potentially lethal cause of atypical genitalia. Congenital adrenal hyperplasia (CAH) is the most common cause of 46,XX DSD. In all situations of adrenal insufficiency, hormone replacement therapy is mandatory and must be promptly started [150].

Sexual hormone treatment during infancy and childhood must be deemed to be critical, as hormone therapy induces irreversible effects. In patients with micropenis, T therapy may be proposed (see the micropene chapter). In patients with 5-alpha reductase deficiency, local DHT therapy has been shown to be effective in increasing the size of the penile shaft.

If the patient’s gonads do not produce enough or indeed any hormones or after a gonadectomy has been performed, sexual hormone treatment become necessary [151] at the appropriate time, as already detailed in the Part 1 of these guidelines [13]. Hormonal induction of puberty is usually performed according to the current practices used for adolescents with pituitary or gonadal deficiency [13, 152]. Clinical studies of hormonal induction of puberty in patients with DSDs have not been conducted so far. The kind of hormone treatment provided should respect the patient’s request. The choice of timing and hormone treatment should be made individually (e.g. in case of uncertainty regarding gender identity). (Patho)physiological aspects must be considered when choosing a hormonal substitution. Refusal of hormone substitution by a patient needs to be discussed, as the procedure is regarded as critical in preventing adverse events. An increase in hormone production during puberty can lead to a change in the child’s phenotype. This might differ from the gender role in which the child/adolescent has lived up to this point. To gain time to think about their continuing gender identity, further development can be stopped by offering GnRH-analogue. In these cases, reference can be made to the guidelines of gender incongruent persons [153–157].

The role of surgery in the management of patients with DSDs is changing, with the advocation that any surgical intervention in neonates and infants that leads to irreversible changes should be done with the utmost caution. Ideally, surgical interventions should construct cosmetically appealing and well-functioning genitalia and preserve fertility in the best possible way. Although great progress has been made in this direction, often this goal is still difficult to achieve. The few publications on long-term outcomes are all retrospective reports and deal with small numbers of patients in whom surgery was often performed more than 20 years ago [158–160]. Surgical procedures have become much more precise and aim at preserving important anatomical and functional structures, although this does not guarantee better results because long-term data of modern procedures will only be available in 10–15 years. Surgical efforts to ‘cure’ patients with DSDs have been detrimental to many individuals in the past. Many individuals with a DSD have complained forcefully about the surgical procedures used. A moratorium on genital surgery in infancy has been proposed by advocacy groups. Currently, the prevailing thought (and legislative recommendations in some countries) claims that specific surgical procedures should be postponed until the individuals can make their own decision about the type of surgical intervention. Only in cases of vital harm and a strictly somatic functional or vital medical indication surgical interventions may be performed on minors [161, 162]. However, some parents of children with CAH, as well as several patients with CAH, do not agree with this rigorous recommendation. Studies confirm that most CAH patients identify themselves as female and do not regret, if they underwent feminization surgery during early childhood [163, 164]. If and when the surgery is decided, it should be performed by an experienced surgeon [165–167]. The surgeon has a particular responsibility to provide realistic and honest information about the possible outcomes of the surgery and the available options (not only surgical). The parents and/or the patient should be informed about post-operative care and possible complications and should be offered regular follow-up. The various forms of XY and X/XY DSD have a variable risk of developing a gonadal germ cell cancer. The risk is much higher in men and women with gonadal dysgenesis, specially forms that arise from early gonadal differentiation defects, such as mutations in SRY and WT1, than in individuals with ovotesticular DSD or with hormone synthesis or action disorders (for example, androgen insensitivity) [168–170]. Overall, the risk of tumorigenesis is also increased by the ectopic position of the gonads which may make the detection of malignancies of abdominal gonads more challenging during early childhood by ultrasound or magnetic resonance imaging. In the absence of reliable tumor markers or imaging technologies for early detection of precursor lesions, it seems prudent to consider gonadal biopsies to exclude the presence of germ cell neoplasia in situ or gonadoblastoma in most individuals with 46,XY or 45,X/46,XY DSD with retained gonads. Given that the age of distribution for testicular germ cell cancers is well established, such biopsies are best performed in late adolescence [171–173]. Because a patient’s own gonads might have a residual function, and because the removal of the gonads is an irreversible intervention affecting the patient’s personal development, it must be considered very carefully, if and when this must be done. If individuals with high tumor risk retain their gonads into adulthood, regular morphological and functional screening must be guaranteed. If gonadectomy is recommended, the possibility of cryoconservation, if vital sperms are found, should be discussed with the patient [174, 175]. In the last few decades, there has been an increasing awareness of the transition to adult-oriented health care in adolescents and young adults with a chronic illness. Few studies have been conducted in adolescents with DSDs during transition, and there is a need for guidelines to manage the transition of these patients. Patients frequently experience difficulties in accessing specialized medical care in adulthood, resulting in loss to follow-up affecting the patients’ physical and psychological health as well as quality of life. Clinical features and long-term outcomes are highly variable in most DSD conditions. Challenges in the care of DSD patients in adulthood are optimization of fertility potential, hormone replacement therapy and sexuality. A good transition process requires close interaction of pediatric with adult endocrinologists with the involvement of urologists, andrologists or gynaecologists, and psychologists [126, 127, 176].

#### Remarks

The foremost objective in managing a newborn with DSD is to identify or exclude adrenal insufficiency, as it necessitates immediate, life-saving hormone replacement therapy. Caregivers, and eventually the patient, must be thoroughly educated on managing the condition and preventing or treating potential adrenal crises. In recent decades, approaches to the medical and surgical management of individuals with atypical genitalia have evolved significantly. In some countries, such interventions have been restricted or deferred until the individual can provide informed consent. This topic remains the subject of ongoing debate, with differing perspectives even among patients and advocacy groups. In confirmed cases of gonadal insufficiency, hormone replacement therapy aligned with the individual’s gender identity should be initiated in accordance with established protocols for hypogonadal patients. Special consideration must be given to gonads at increased risk for neoplastic transformation. In such cases, prophylactic gonadectomy is indicated, with the potential option of preserving unaffected gonadal tissue when feasible.

## Micropenis

### Epidemiology

Isolated micropenis is defined as a stretched penile length (SPL) 2.5 standard deviations below the mean for age group (Table 5) without the presence of any other penile anomalies such as hypospadias [177, 178]. Minor ethnic differences in SPL have been reported [179, 180], though these are less evident in infants and young boys, and the growing immigrant populations in some Western countries should be considered [181]. Micropenis is a different condition respect to buried, webbed, trapped or diminutive penis, although all of them are included into the term “inconspicuous” [182, 183]. The incidence of micropenis is known to be 1.5 of 10,000 male newborns in United States [184]. According to the definition, approximately 0.8% of the male general population can cary this condition, although the isolated idiopathic or nonspecific 46,XY DSD form, which we will discuss in this section, constitutes about 25% of these forms [185].

**Table 5:** Streched penile lenght [SPL cm; mean +/-SD (mean-2.5 SD)] in normal males according to age for samples of North American, European and Asian Populations

Age	Asian population [377]	European population [378]	American population [379]
Newborns 30 wks*	-	-	2.5 ± 0.4 (1.5)*
Newborns 34 wks*	-	-	3.0 ± 0.4 (2.0)*
0–5 mos.	-	-	3.9 ± 0.8 (1.9)
6–12 mos.	-	-	4.3 ± 0.8 (2.3)
0–1	4.1 ± 0.4 (3.1)	3.5 ± 0.5 (2.4)	
1–2	4.2 ± 0.4 (3.2)	3.7 ± 0.5 (2.3)	4.7 ± 0.8 (2.6)
2–3	4.3 ± 0.4 (3.3)	3.9 ± 0.5 (2.9)	5.1 ± 0.9 (2.9)
3–4	4.4 ± 0.4 (3.4)	4.0 ± 0.6 (2.5)	5.5 ± 0.9 (3.3)
4–5	4.5 ± 0.5 (3.2)	4.3 ± 0.7 (2.5)	5.7 ± 0.9 (3.5)
5–6	4.6 ± 0.5 (3.3)	4.4 ± 0.6 (2.9)	6.0 ± 0.9 (3.8)
6–7	4.7 ± 0.6 (3.2)	4.5 ± 0.6 (3.0)	6.1 ± 0.9 (3.9)
7–8	4.8 ± 0.6 (3.3)	4.7 ± 0.7 (2.9)	6.2 ± 1.0 (3.7)
8–9	4.9 ± 0.7 (3.1)	4.7 ± 0.7 (2.9)	6.3 ± 1.0 (3.8)
9–10	5.1 ± 0.8 (3.1)	4.7 ± 0.7 (2.9)	6.3 ± 1.0 (3.8)
10–11	5.4 ± 0.8 (3.4)	4.8 ± 0.7 (3.0)	6.4± 0.1 (3.7)
11–12	5.7 ± 1.0 (3.2)	5.1 ± 0.9 (2.9)	-
12–13	6.2 ± 1.2 (3.2)	5.9 ± 1.4 (2.4)	-
13–14	6.8 ± 1.4 (3.8)	7.1 ± 1.6 (3.1)	-
14–15	7.6 ± 1.4 (4.1)	8.0 ± 1.3 (4.7)	-
15–16	8.2 ± 1.5 (4.4)	8.7 ± 1.2 (5.7)	-
16–17	8.8 ± 1.6 (4.8)	9.0 ± 1.1 (6.2)	-
17–18	9.5 ± 1.6 (5.5)	9.1 ± 1.1 (6.3)	-
18–19	10.2 ± 1.7 (5.9)	9.1 ± 1.1 (6.3)	-
19–20		9.5 ± 1.1 (6.7)	-
Adults			13.3 ± 1.6 (9.3)

* [380]

### Pathophysiology

The human penis develops from the genital tubercle (GT) visible by 5–6 weeks of gestation [186, 187]. From 8 weeks, maternal hCG stimulates testosterone production from fetal Leydig cells. Testosterone, converted to dihydrotestosterone (DHT), directs penile differentiation, a process largely complete by 12 weeks, when the GT forms the glans, genital folds form the penile shaft, and genital swellings merge to create the scrotum. During the second and third trimesters, penile growth is driven by fetal androgens, regulated by fetal pituitary gonadotropins, with the penis increasing almost 2 cm between weeks 16 and 38 [188]. Therefore, true micropenis reflects a hormonal defect occurring after 12 weeks of gestation.

Congenital micropenis may indicate a range of endocrine or syndromic conditions (Table 6) that require evaluation. After excluding hormonal deficiencies, a minority of patients remain undiagnosed and may be classified as carriers of idiopathic or nonspecific 46,XY DSD [189, 190].

**Table 6:** Conditions associated with micropenis

**Hypothalamo pituitary disorders (Hypogonadotropic hypogonadism):**
-Isolated
-Combined with other pituitary hormone deficiency
-Syndromic conditions: Prader-Willi syndrome; Bardet-Biedl syndrome; Laurence-Moon syndrome; Charge syndrome; Silver Russel syndrome; Rud syndrome
-Isolated Growth Hormone or IGF-1 deficiency
**Gonadal disorders (Hypergonadotropic hypogonadism):**
-Anorchia/Vanishing testes syndrome
-Disorders of gonadal development: Sex chromosome mosaicism; Partial gonadal dysgenesis
-Disorders of androgen synthesis: 3-beta-hydroxysteroid dehydrogenase deficiency; 17-beta-hydroxysteroid dehydrogenase deficiency; 17,20-lyase deficiency isolated or combined with 17-hydroxylase deficiency; 5-alfa-reductase deficiency
-Disorders of androgen action: Partial androgen insensitivity syndrome
-Chromosomal/genetic syndromes: Klinefelter (47XXY) and multiple X syndromes (48XXXY), Downs syndrome, Noonan syndrome, Robinow syndrome, CHARGE syndrome
-Penile agenesis (aphallia)
**Miscellaneous:**
-Maternal use of antifungals
-Environmental endocrine disruptors
-Nonspecific 46 XY DSD or idiopathic micropenis (i.e., cases with no evidence of endocrine or genetic abnormalities)

### Clinical picture

Penile length is measured along the dorsum with the penis stretched, from the pubic symphysis to the glans tip, using a rigid ruler [178, 180, 189]. Penile girth should be recorded at both the base and coronal level [191]. Corpora cavernosa can usually be palpated, even if hypoplastic; the scrotum is often small, and testes may be small or incompletely descended. Stancampiano et al. [189] reported the median/mean SPL values across childhood and adolescence for different ethnic backgrounds, and Table 5 shows mean − 2.5 SD values for country-specific norms. According to international guidelines, micropenis is generally defined as < 2 cm at birth and < 4 cm after 5 years of age [190].

### Diagnostic evaluation

#### Recommendations and suggestions

**R3.1** We recommend that infants with congenital micropenis need to be considered for a complete clinical and endocrine evaluation by a specialized multidisciplinary team to exclude all hormonal or syndromic causes of the condition (1, ⊕⊕⊕⊕).

**R3.2** We recommend that newborns with isolated micropenis, regardless of subsequent diagnostic investigations, carry out a hormonal work-up during the first 2 days of life or during the minipuberty window (3 to 6 months of life), assessing the basal pituitary-gonadal function (Testosterone, LH, FSH, Inhibin B and AMH serum levels) (1, ⊕⊕⊕○).

**R3.3** We recommend that children/adolescents with isolated idiopathic micropenis have an accurate measurement of the stretched penile length, palpation of the corporeal bodies with measurement of girth/diameter, and evaluation for cryptorchidism (1, ⊕⊕⊕⊕).

**R3.4** We recommend reviewing the patient with isolated idiopathic micropenis at least once in the prepubertal period and regularly from the time of expected puberty on to evaluate the development of sexual characteristics and possible supportive hormonal therapy (1, ⊕⊕⊕⊕).

**R3.5** We suggest that in case of peri-pubertal presentation, the patient be examined in collaboration between pediatric and adult endocrinologists/andrologists, to familiarize the patient to the transition phase and long-term follow-up (2, ⊕⊕○○).

**R3.6** We suggest that the family and/or the patient with micropenis should already be offered competent psychosocial attendance by a professional familiar with problems of the genito-urinary tract (2, ⊕⊕○○).

#### Evidence

Since isolated micropenis can be the marker of a wide range of endocrine alterations that include the hypothalamic-pituitary-gonadal axis or DSD [189], the evaluation of an expert multidisciplinary team is essential for diagnostic and therapeutic orientation (see the chapters on “Pubertal Delay” and “DSD”).

A sample collected during the first 2 days of life or during the minipuberty window (3 to 6 months of life), as above mentioned, is the unique temporal opportunity to highlight any physiological rise in gonadotropins and testosterone to evaluate the functioning of the pituitary-gonadal axis [71, 192, 193].

The penile length must be measured on a stretched penis by a trained expert since this is the measure that is strongly related to erected penis length [191]. The measurement of penile circumference and diameter should be performed by a flexible centimeter and a caliper, respectively.

The transition process of boys with isolated idiopathic micropenis allows for the establishment of a long-term follow-up useful for evaluating the long-term outcomes of those who have or have not undergone therapies. It is unclear whether the presence of isolated idiopathic micropenis itself leads to long-term consequences apart from psychosocial distress in parents and young adults. In fact, studies investigating aspects of self-esteem, sexual function, and fertility in young adults with this condition are scarce and dated [194]. Psychosocial support should always be offered when available and an external consultant should be identified when it is not [195].

#### Remarks

When SPL is measured in the newborn during the first 12 h of life the length may be reduced by approximately 10% and should be reassessed [196]. The SPL inter- and intra-observer variation for trained personnel has been reported as 1 SD of 0.34 cm and 0.18 cm, respectively [197]. A standard karyotype does not allow to highlight possible hidden mosaicisms thus a higher number of metaphases (e.g., *n* = 100) is always needed to improve accuracy [198]; karyotype may be preceded whenever possible by a fluorescence in situ hybridization (FISH) for the sex-determining region on the Y chromosome (SRY) for a faster exclusion of a DSD condition [199].

In case of micropenis belonging to a patient with a documented diagnosis of delayed puberty, an appropriate management should be settled, according to Part-1 of these guidelines [13].

### Therapeutic management

#### Recommendations and suggestions

**R3.7** We recommend that isolated idiopathic micropenis discovered early in life is treated by a pediatric endocrinology center (1, ⊕⊕○○).

**R3.8** We suggest that patients with idiopathic micropenis and established androgen sensitivity, may be treated with testosterone/DHT therapy during the first 6 months of life or later in childhood/adolescence to stimulate the growth of the penis (2, ⊕⊕○○).

**R3.9** We recommend against surgical therapy before adulthood, postponing this option in adult life (1, ⊕⊕○○).

#### Evidence

There is no consensus on the age, dosage, method of administration, or duration of T or DHT therapy in boys with isolated micropenis. A regimen of intramuscular depot-testosterone enanthate 25 mg administered monthly for 3 months has been reported to show an increase in penile length of over 100% in prepubertal boys with isolated micropenis. However, the study does not provide information on the long-term durability of the results achieved [200]. The same results with Te enanthate at the same dosage are reported also during minipuberty [22]. Arisaka et al. also documented a significant increase in SPL in 50 prepubertal boys treated with T cream 5% (10 mg/daily applied directly to the penis) for a duration of 30 days. The study documents the systemic absorption of transdermal T, although this does not appear to have significant negative effects on bone metabolism while Xu D et al. found that short term (6 months) and local application of DHT at low doses (0.1–0.3 mg/kg/day) in patients with micropenis could accelerate penile growth effectively without evident side effects [201, 202]; however, precautions still need be taken due to the paucity of long term data and the lack of ideal DHT dosage.

A recent randomized study on 49 patients compared the efficacy of transdermal DHT (5 mg/daily for 5 weeks with 1 or 2 repetitions if necessary) and T enanthate (50 mg i.m. monthly for 3 months, with 1 repetition if necessary) in treating isolated idiopathic micropenis in children and adolescents (mean age 9.7 ± 4.4years). The study suggested a superiority of transdermal DHT compared to injectable exogenous T in the treatment of idiopathic micropenis, although both showed significant penis enlargement (mean penile size increment 2.82 vs. 1.87 cm, respectively) [203]. Based on the available evidence we thus suggest using T enanthate 25 mg i.m. in prepubertal children once a month for 3 months, repeatable only once after 3–6 months; or transdermal DHT (5 mg/daily for 6 weeks, repeatable 1–2 times). No evidence supports the use of hormonal therapies after puberty.

#### Remarks

Precautions still need be taken due to the paucity of long-term studies and the lack of ideal T and DHT dosage [189]. Moreover, T enanthate and DHT gel is off label for treatment during pediatric age (0–18 yrs.), at least in Italy, and an informed, signed consent need to be obtained by parents/legal representative of the minor. Furthermore, it should be considered that DHT is not available in all countries.

There is still uncertainty about the optimal age of therapy with androgens for maximal effect; given that tissue AR expression is high in early infancy, it would seem appropriate to use androgens at that point, but it remains unclear whether such early use of androgens has any benefit on penile length in adulthood [204].

In addition to ongoing psychological support for the family and patient, the involvement of the adult endocrinologist/andrologist in the transitional period will help to explain the adolescent/young adult on the pro and cons of other future surgical options. Open doctor-patient communication regarding expectations, specific risks, benefits, and alternatives is of paramount importance in enabling the best possible results in this sensitive field [205].

During the transition process the adolescents/young adults must be counseled by expert personnel and advised with extreme caution about surgical options due to uncertain functional efficacy and the high rate of complications among patients with micropenis [190].

## Hypospadias

### Epidemiology

Hypospadias consists of an arrest in normal penile/urethral development with hypoplasia of the tissues forming the ventral side of the penis responsible of an ectopic meatus of the urethra. The earlier this process arrests, the more the form is proximal and severe [206]. Hypospadias is one of the most common congenital anomalies, although there is wide variation of prevalence according to countries and ethnicity (13.8 to 40/10,000), and there are conflicting data on the recent trends of prevalence [207, 208].

### Pathophysiology

Male and female genital development diverges from the indifferent stage around 8 weeks’ gestation under androgen influence. In normal males, the urethral groove fuses proximally to distally to form the tubular urethra, a process completed by ~ 17 weeks along with fusion of the ventral foreskin and straightening of the early penile curvature [209]. In hypospadias, disruption of urethral canalization or fusion results in an ectopic urethral meatus along the ventral shaft [206]. The pathogenesis is often unknown, but disrupted androgenic signaling, combined with genetic (Table 7) and environmental (Table 8) factors, is considered the main cause [210–215]. Proximal hypospadias, particularly when associated with cryptorchidism or micropenis, is more likely to have a genetic etiology. Commonly associated abnormalities include ventral penile curvature (chordee), hooded or incomplete prepuce, and hypoplastic corpora spongiosum [206].

**Table 7:** Genes potentially involved in hypospadias. Most of them are more frequently associated with other genital or multiorgan malformations [381]

Gene	MIM
**Indifferent stage or early embrionic**	
*WT1*	*607,102
*NR5A1 (SF1)*	*184,757
**Early patterning**	
*BMP4*,* BMP7*	*112,262, *112,267
*HOXA4*,* HOXB6*,* HOXA13*	*142,953, *142,961, *142,959
*FGF8*,* FGF10*	*600,483, *602,115
*FGFR2*	*176,943
*IRX5*	*606,195
*IRX6*	*606,196
*EYA1*	*601,653
*DGKK*	*300,837
*ZEB1*	*189,909
**Masculinization**	
*AR*	*313,700
*SRD5A2*,* SRD5A1*	*607,306, *184,753
*SRY*	*480,000
*SOX9*	*608,160
*FKBP4*	*600,611
*HSD3B2*	*613,890
*HSD17B3*	*605,573
**Other**	
*ESR1*,* ESR2*	*133,430, *601,663
*ATF3*	*603,148
*MAMLD1 (CXORF6)*	* 300,120
*MID1*	*300,552
*INSL3*	*146,738
*BNC2*	*608,669

**Table 8:** Environmental factors potentially interfering with male genitals formation. (Modified from [214])

Factors frequently investigated	Factors not frequently investigated
*Factors consistently associated with hypospadias*	
LBW/SGA	
Placental insufficiency	
Maternal Hypertension	
Pre-eclampsia	
Maternal intrauterine DES exposure	
*Factors associated with hypospadias in most studies*	*Factors that seem to be associated with hypospadias*
ICS	Paternal subfertility
Prolonged TTP	Absence of nausea and vomiting in early pregnancy
High maternal BMI	Bleeding during pregnancy
Primiparity	Complications during labour
Multiple pregnancy	Maternal use of antihypertensive
Pre-existing maternal diabetes	
Maternal use of antiepileptics	
*Factors showing inconsistent results*	*Factors showing inconsistent results*
Preterm	Early age at menarche
Iron supplementation	Maternal thyroid disease
Maternal age	Fever in first trimester
Maternal vegetarian diet	Progestin/progestogens for threatened abortion
Maternal fish consumption	Paternal heavy metal exposure
Maternal and paternal exposure to pesticides	Living in rural or urban areas
Maternal occupational exposure to endocrine disruptors, heavy metals, phthalates	
Maternal serum levels of PCB’s	
Seasonal trend	

### Clinical picture

**Fig. 1:**
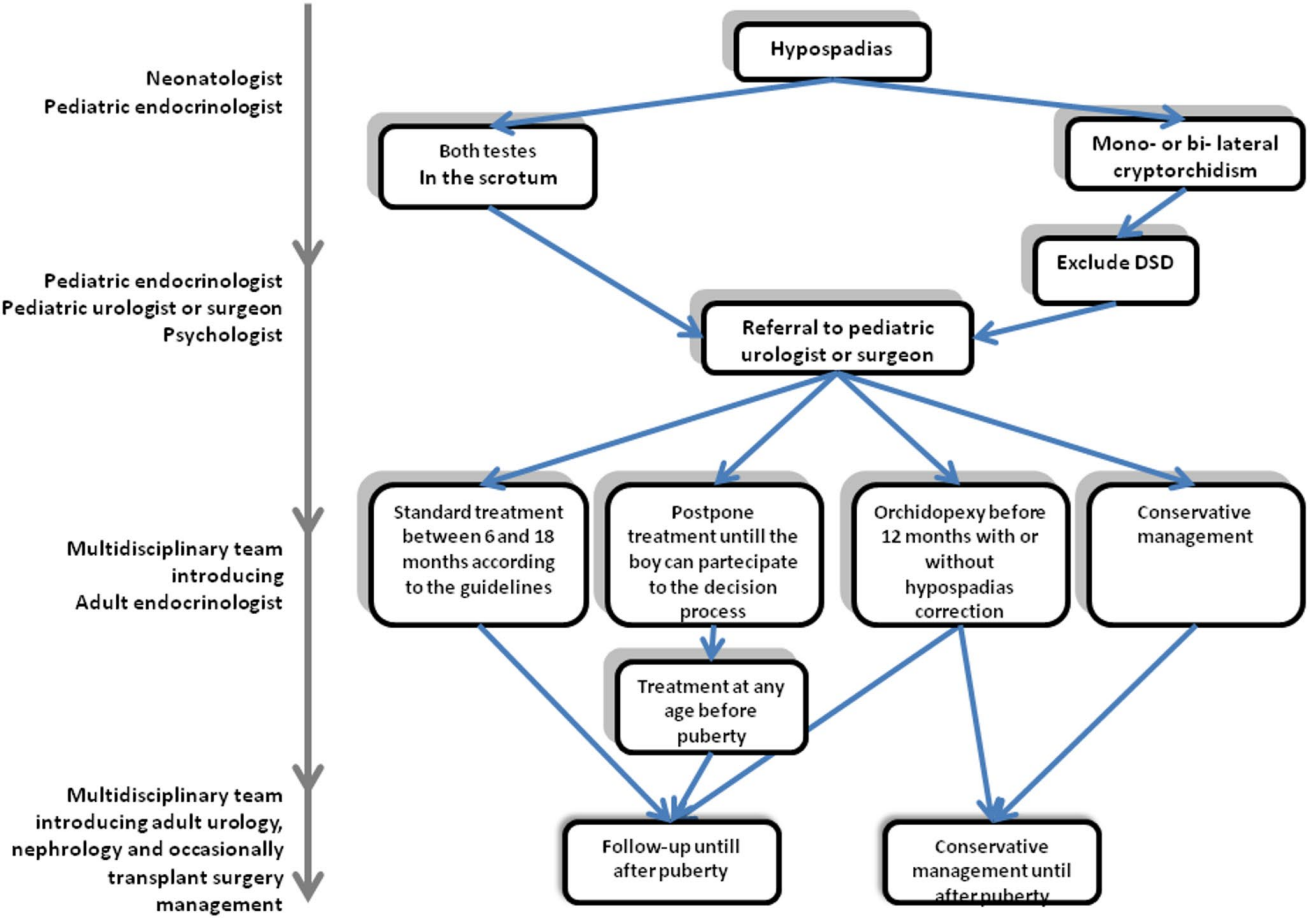
Proposal algorithm for referral and treatment of hypospadias. (Modified from Ref. [235].

Evaluation of hypospadias includes family history, detailed genital examination, and assessment of other congenital anomalies [216]. Examination should record SPL, penile curvature, foreskin development, and testicular position (Fig. 1). Preoperative classification is typically based on urethral meatus location: anterior (glandular/subcoronal), middle (distal/proximal/midshaft), and posterior (penoscrotal, scrotal, perineal) [217]. A more precise characterization also considers foreskin appearance and degree of curvature (Table 9) [206].

**Table 9:** Classification of hypospadias based on physical findings. (Modified from [206])

Type	Frequency (%)	Urethral location	Penile curvature^	Foreskin appearance	Management	
Partial forms (incomplete presence of hypospadias)	10	Normal: with a urethral pit or ectopic urethra on the distal glans	Normal	Normal to mild asymmetric ventral deficiency	No surgical correction or evaluation needed	
Standard (normal glans: MD* ≥ 1.4 cm)	40	Distal: on glans or coronal margin	Normal to mild	Ventral deficiency with a dorsal covered appearance	Surgical correction is an option; families may choose observation; no other evaluation	
	25	Proximal: on penile shaft, at the penoscrotal connection, or within the scrotum	Moderate		Surgical correction is suggested; no further evaluation	
Severe (abnormally small glans: MD* < 1.4 cm)	20	Scrotal or perineal	Severe	Ventral penile foreskin tethering (short urethral plate) or fusion of the foreskin to the scrotum	Surgical correction required (usually two stages procedure); endocrine/genetic evaluation to detect DSD	
Us with intact prepuce		Large urethral opening at the coronal margin	Normal	Normal	Correction usually performed on family preference; no other evaluation	

**^**Penile curvature is assessed when the penis is erect and classified as normal (0 to 15 degrees), mild (15 to 30 degrees), moderate (30 to 75 degrees), and severe (> 75 degrees); *MD: maximal diameter

Quantitative scoring systems facilitate objective description of external genitalia in neonates and infants. A non-binary version of the External Masculinization Score (EMS) [117], the European External Genital Score (EGS), has been validated in multicenter studies (Table 4) [128]. The anogenital distance (AGD) correlates with prenatal androgen exposure [218], with males showing a higher AGDl/u ratio (0.49 ± 0.1) than females (0.39 ± 0.1), and EGS correlates with AGDl/u in males [128]. Such standardized metrics improve clinical assessment, surgical planning, and multicenter research.

### Diagnostic evaluation

#### Recommendations and suggestions

**R4.1** We recommend evaluating hypospadias by detailed genital examination and classifying its severity by the physical findings reported in Table 3 (1, ⊕⊕⊕○).

**R4.2** We suggest evaluating the commonly used anthropometric indicators such as external genitalia score (EGS), anogenital distance (AGD) and penoscrotal distance (PSD) in neonates and children carrying hypospadias (2, ⊕⊕○○).

**R4.3** We recommend that in proximal and complex hypospadias, further diagnostic evaluation is performed, such as ultrasonography of the urinary tract and internal genital organs to detect other genito-urinary malformations (1, ⊕⊕⊕⊕).

**R4.4** We recommend genetic testing in patients with scrotal or perineal hypospadias, especially if associated with cryptorchidism and micropenis because they are at high risk of Differences of Sex Development (DSD) (1, ⊕⊕⊕○).

#### Evidence

Several preoperative classification systems have been proposed to assess hypospadias severity based on position of the urethral meatus opening [218]. However, they lack precision and do not reliably consider the whole penile anatomical disruptions. From this aspect, the classification proposed by Baskin L, which we report in Table 3, considers the main anatomical aspects involved in the severity of hypospadias and offers relevant suggestions for the surgical or non-surgical management of this condition [206].

The current refined classification of hypospadias relies mainly on the quality of the urethral plate, the position of the urethral meatus, glans size and the degree of curvature. However, this can only be determined during surgery and there is still significant subjectivity between evaluators. If the postoperative classification can be described and predicted preoperatively based on local anatomical data, it will be of great significance for surgeons to design a proper surgical plan and preoperatively communicate with family members. He et al. has defined the anatomical abnormalities of hypospadias before puberty using current commonly used anthropometric index data to predict postoperative diagnostic classification. They found that anogenital distance (AGD) and penoscrotal distance (PSD) have a favorable predictive value for midshaft and proximal hypospadias [219]. Over 20% of boys with hypospadias are also known to have other extra-genital congenital conditions [220, 221]. In these cases, endocrinological evaluation is advised to exclude DSD (see DSD chapter), especially in case of concomitant unilateral or bilateral undescended testis [218].

#### Remarks

Severe hypospadias can masquerade as atypical genitalia warranting an evaluation for a disorder of sex development (see DSD chapter). The incidence of DSD is significantly increased in proximal or complex hypospadias and further assessment needs to be done to individuate patients with these conditions (pelvic ultrasound, karyotype, hormonal assessment and serum electrolytes) [222]. In cases where there are multiple major birth defects, further evaluation to detect an underlying syndrome or genetic defect must be performed [223, 224].

To evaluate the AGDs and the PSD measures, the TIDES method places the infant in a supine position with the lower half of the body exposed and the legs lifted in a froglike posture (with a 60–90° angle from the torso at the hip) and knees pulled back towards to shoulders [225]. AGDl (AGDlower), is measured from the center of the anus to the base of the labioscrotal border; AGDu (AGDupper), is measured from the center of the anus to the anterior base of the genital tubercle [128]. PSD measurement is based on skin fold boundaries to reflect the distance from the front, back, left and right (12, 9, 3 o’clock) of the root of penis to the border of scrotum [219].

### Therapeutic management

#### Recommendations and suggestions

**R4.5** We recommend urologic referral and repair only for patients with hypospadias who are at-risk for voiding and/or sexual dysfunction (1, ⊕⊕○○).

**R4.6** We recommend against performing circumcision in the newborn period for patients with asymmetric foreskin due to abnormal development on the ventral aspect of the penis (1, ⊕⊕⊕○).

**R4.7** We suggest that hypospadia correction should preferably be performed between 6 and 18 months of age in full-term, healthy infants (2, ⊕⊕⊕○).

**R4.8** We suggest tubularized incised plate urethroplasty (TIPU) or dorsal inlay graft urethroplasty (DIGU) as options for repairing of standard hypospadias, although, at present, no technique has demonstrated to be better (2, ⊕⊕○○).

**R4.9** We suggest that a two-stage hypospadias repair should be used for the most severe forms of hypospadias, despite evidence on the best surgical technique is lacking (2, ⊕⊕○○).

**R4.10** We suggest preoperative hormonal therapy only for proximal hypospadias or hypospadias with microphallus (2, ⊕⊕○○).

**R4.11** We recommend that the adolescents/young adults with hypospadias who underwent childhood repair should be adequately involved in a long-term follow-up to detect any physical/psychosocial problem that may emerge during the transition from adolescence to adulthood (1, ⊕⊕⊕○).

**R4.12** We suggest using validated objective scoring systems to assist in evaluating the functional and cosmetic outcome of patients who underwent surgical repair (1, ⊕⊕⊕○).

#### Evidence

Surgical repair is not necessary for patients with mild defects (partial or standard distal forms of Table 3). This conservative approach is supported by a report of 56 adults with unrepaired hypospadias including 44 with a mild form who were satisfied with the appearance of their penis, voided in standing position and did not have infertility associated with the abnormal position of the urethral meatus [226].

Urologic referral and potentially surgical repair are indicated for patients who are at risk of: (a) significant deflection of the urinary stream; (b) inability to urinate from a standing position; (c) erectile dysfunction due to penile curvature leading to intercourse difficulties; (d) fertility issues due to sperm deposition difficulties; (e) concern about developmental problems based on the appearance of the penis (Baskin L, 2025, www.UpToDate.com).

The choice of the age range 6–18 months for primary hypospadias repair arises from the awareness that emotional, cognitive, and body image development may be affected by both the genital anomaly and the reconstructive surgery. These psychological factors matter, as the child’s reaction to both the surgery and the anesthetic trauma varies strongly with age. Postoperative behavioral problems such as aggressive or regressive behavior, night terrors, and anxiety may be more common at certain ages, particularly at 1 to 3 years of age. The period from 6 to 15 months is a relatively good time for surgery from the viewpoint of emotional development, provided parent/child separation is minimized [227]. However, age at surgery is not a risk factor for urethroplasty complication in pre-pubertal tubularised incised plate urethroplasty (TIP) repair [228]. On the contrary, complication rate after primary TIP repair was 2.5 times higher in adults than in the pediatric group according to a prospective controlled study [229].

Two systematic reviews and metanalysis assessed whether dorsal inlay graft urethroplasty (DIGU) had any additional advantages over standard tubularised incised plate urethroplasty (TIPU) repair in children with primary hypospadias. There is not enough evidence to suggest that either technique offers more favorable outcomes. Until more robust randomized clinical trials (RCTs) exist, decisions regarding the appropriate repair should be based on the surgeon’s experience and outcomes [230, 231].

The systematic review of Borkar et al., based on RCTs, provides evidence supporting the use of double dartos flap (DDF) over single dartos flap (SDF) in hypospadias repair, particularly in distal hypospadias using the TIPU procedure [232].

Hypospadias repair following neonatal circumcision in the absence of preputial skin is a challenging reconstruction. The reoperation rate in a paper of Erzberg et al. was 30%, like reoperative hypospadias surgery. Parents of newborns diagnosed with hypospadias should be encouraged to refrain from pre surgical routine neonatal circumcision [233].

There is no consensus on the best surgical approach to correcting severe hypospadias. Despite more than 250 different techniques for hypospadias repair, successful outcome depends mainly on the surgeon’s skills and the availability of appropriate tissue [234, 235]. A recent meta-analysis comparing the outcomes of single stage (foreskin pedicled tube) versus two stage (foreskin free graft, FFG & foreskin pedicled flap, FPF) repair for proximal hypospadias in the last decade found that two-stage repair of proximal hypospadias had significantly less complications compared to single stage repair [236]. Among two-stage repairs specific complications were significantly less for FFG, although total complications were not significantly different from that seen with FPF. The results of two-stage repairs improved with higher case load supporting the concept of dedicated hypospadias centres [236].

Chua et al. systematically evaluated the effect of preoperative hormonal therapy (PHS) on postoperative complications rates following hypospadias repair. Pooled effect estimates with moderate quality of evidence from three RCTs suggested that significant lesser postoperative complications occurred among patients exposed to PHS (RR 0.36, 95% CI 0.20–0.65) [237].

**Fig. 2:**
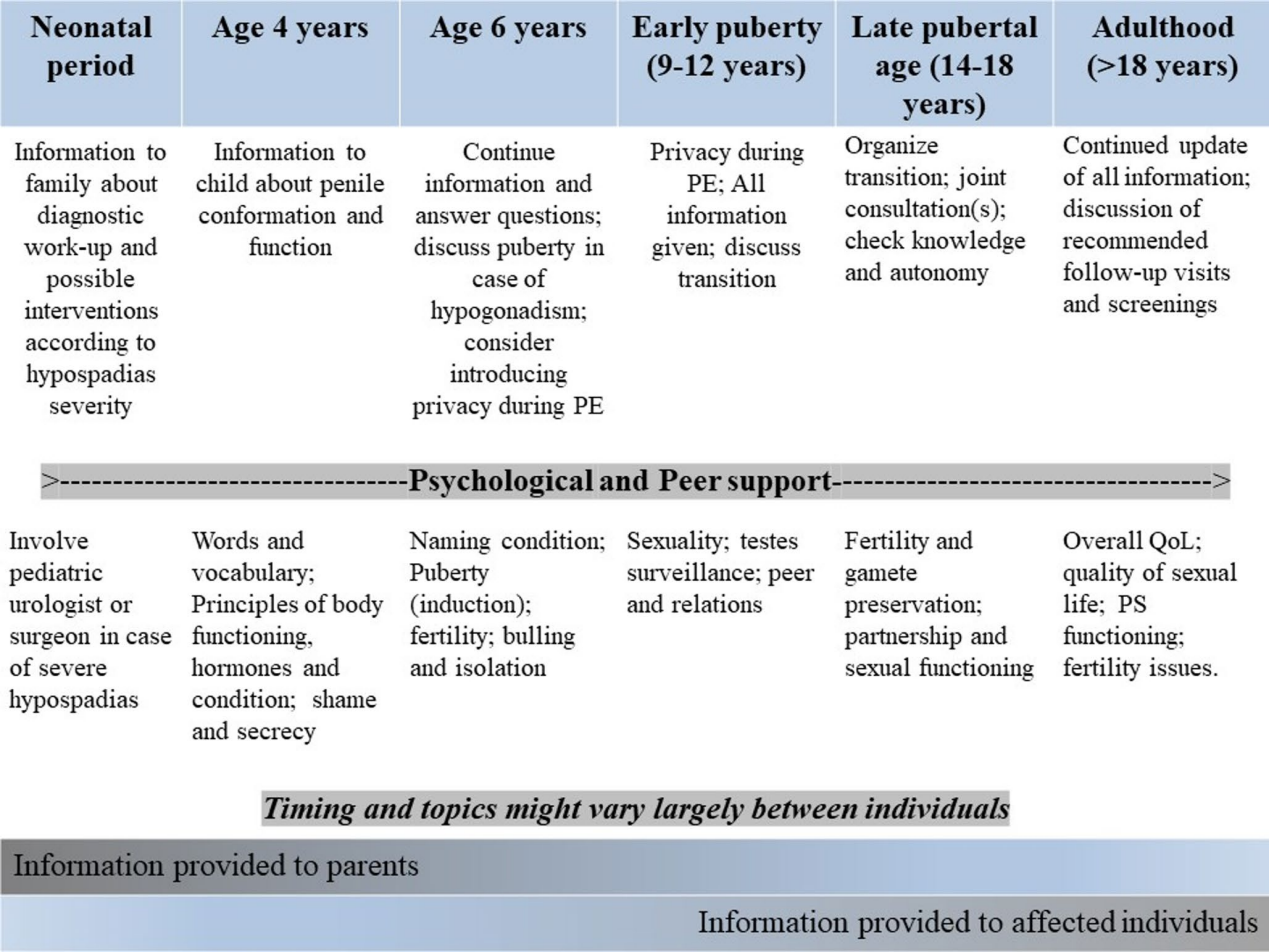
The present figure reproduces the structure and partially modifies the content of fig. 2 in reference [106], after adaptation to hypospadias condition. Multidisciplinary care and data collection start at diagnosis and maintain throughout the individual’s life. The focus of the information process gradually shifts from the parents to the affected child. Psychological and peer support are key elements at all ages. With appropriate individual adaptations, children should be informed of their conditions at an early age. Some of the suggested themes to be discussed by the team members are shown in boxes at the upper part of the figure and the lists of some important topics within these themes are represented in boxes at the lower part of the figure. PE, physical exam; PS, psychosexual; QoL, quality of life

Surgery for hypospadias remains challenging and complications may occur lifelong: early after surgery, during puberty due to fast growth, and in adulthood. The functional objectives of surgery are to create a normal urinary stream, normal erections, and normal coitus with a satisfactory cosmesis as well [238]. However, these objectives can be elusive and multiple operations including free graft harvesting have been described for hypospadias repair. As boys reach puberty, despite initial successful surgical treatment of posterior hypospadias, penile development can result in new functional concerns such as fistula enlargement, worsening penile curvature, and poor cosmesis [239–241]. Patients with hypospadia should be routinely instructed to return for follow up after puberty. At that point, these teenagers should be reassessed with detailed physical examination and uroflowmetry. An open discussion of all available relevant medical data, including progressive information on any until now insufficiently communicated aspects of the condition, is crucial (Fig. 2) [106]. In addition, completing the Hypospadias Objective Penile Evaluation (HOPE) questionnaire or other validated scales to assess the functional, social and psychological outcomes will allow for a more objective and generalizable evaluation of the repair [242–245].

The network meeting “Lifelong Posterior Hypospadias” was organized to identify what is currently missing in the lifelong treatment of posterior hypospadias, to improve care, quality of life, and awareness for these patients. The participants concluded that patients should be more involved in defining desired treatment approach and outcome measures. For optimal outcome evaluation, standardization of data collection with cooperation between basic researchers from different centers, as well as involving clinicians and patients is necessary [246].

#### Remarks

Surgery of milder forms of hypospadias is an option for some families who want to pursuit reconstruction to improve cosmetic appearance for religious (circumcision) or social reasons. When circumcision is desired by the family, it should be delayed until a freehand circumcision can be safely performed in the operating room later in life (Baskin L, 2024, www.UpToDate.com).

Repeated follow-up and psychological support during childhood/adolescence is of great importance especially for patients with more proximal hypospadias. In fact, a case-control study performed in Sweden to assess a possible negative influence on the psychosocial outcome in adult males with hypospadias showed that “distal” patients responded similarly to controls on several measures, and 70% did not desire additional medical follow-up; on the contrary, there were clear psychosocial differences in patients with mid-shaft and proximal hypospadias that warranted additional support [247].

Teenagers with hypospadias who will be transitioning into an adult urology practice need to have ownership of their condition and be aware of what happened in the past in terms of number of previous surgeries and type of surgical technique used. This type of pediatric patient maturation will allow for a smoother transition to adult specialists, even when their medical records are not available [248, 249].

## Epididymitis and orchitis

### Epidemiology

Orchitis and epididymitis are inflammation of the testis and epididymis, respectively, with or without infection.

Isolated orchitis is rare and usually accompanied by epididymitis, hence the true incidence is unclear [250]; moreover, current literature does not suggest any differential occurrence according to race or religion [250].

The annual incidence of acute epididymitis is approximately 1.2 per 1,000 pre-pubertal boys (mean age = 11 years), and it is approximately 1 per 144 from post-pubertal to transition; about 1/4 of patients in post-pubertal and transition age has recurrence within five years [251, 252].

### Pathophysiology

Orchitis is the unilateral or bilateral inflammation of the testis, usually caused by viral or bacterial infections, while epididymitis is the inflammation of the epididymis and represents the most common intrascrotal inflammatory condition [250–252]. Both can be classified as acute (≤ 6 weeks) or chronic (> 6 weeks).

In children and adolescents, orchitis is usually viral, most commonly due to mumps or rubella, with mumps responsible for most isolated cases; rare cases have been reported after MMR vaccination [250].

Epididymitis typically results from retrograde pathogen ascent, most often bacterial [251, 252] although, non-infectious aetiologies such as vasculitis, autoimmune diseases, or medications (e.g., amiodarone) have been identified [253–258]. In prepubertal boys, epididymitis is generally a post-infectious inflammatory reaction to pathogens like mycoplasma, enteroviruses, or adenoviruses and usually follows a benign course. In postpubertal adolescents and young adults, sexually transmitted infections are the primary cause [251, 252].

### Clinical picture

Epididymo-orchitis presents with gradual onset scrotal pain, sometimes radiating to the lower abdomen. Acute cases develop rapidly and last less than 6 weeks, whereas chronic cases progress more slowly and persist beyond 6 weeks. Symptoms may be associated with lower urinary tract infection or urethritis, including urethral discharge, dysuria, or penile irritation [259–267]. Testicular torsion is the main differential diagnosis and should be promptly considered as indicated below in the specific session.

### Diagnostic evaluation

#### Recommendations and suggestions

**R5.1** We recommend an andrological consultation in case of symptoms and signs of epididymo-orchitis to confirm the clinical diagnosis (1, ⊕⊕⊕⊕).

**R5.2** We recommend performing bilateral scrotal ultrasonography (1, ⊕⊕⊕○).

**R5.3** We suggest for Neisseria Gonorrhoeae and Chlamydia Trachomatis infections, acid amplification test (NAAT), in sexually active patients (2, ⊕⊕○○).

#### Evidence

In mumps, orchitis develops in both pre-pubertal and post-pubertal groups of patients, more rarely in pre-pubertal patients, and in 14–35% of patients in post-pubertal and transition age; the clinical syndrome develops 4 to 8 days after parotitis but can also occur in its absence [250]. Different viruses causing orchitis include varicella-zoster virus, coxsackievirus, echovirus, and cytomegalovirus [250]. Bacterial infections of the prostate and urinary tract infection can cause orchitis. Common causes of bacterial orchitis include *Escherichia coli*, *Klebsiella pneumoniae*, *Pseudomonas aeruginosa*, and *Staphylococcus* and *Streptococcus*. Bacteria causing sexually transmitted infections can also determine orchitis in sexually active males; common pathogens include Neisseria Gonorrhoeae and Chlamydia Trachomatis [250]. Risk factors for epididymitis in pre-pubertal boys include recent urinary tract surgery or instrumentation procedures and anatomic abnormalities, such as posterior urethral valves or meatal stenosis, whereas risk factor in post-pubertal up to transition age include sexual activity, strenuous physical activity, bicycle or motorcycle riding, and prolonged periods of sitting, and epididymitis is most commonly caused by sexually transmitted infection by Neisseria Gonorrhoeae or Chlamydia Trachomatis [251, 252].

#### Remarks

Since sexually transmitted infections can rarely be found in pre-pubertal boys, diagnostic evaluation using NAAT must be guided by the presence of risk factors such as belonging to ethnic groups at risk, signs of abuse, presence of associated urethritis.

### Therapeutic management

#### Recommendations and suggestions

**R5.4** We recommend the use of anti-inflammatory treatment for symptomatic epididymo-orchitis (1, ⊕⊕⊕○).

**R5.5** We suggest for Neisseria Gonorrhoeae infection: ceftriaxone i.m. once plus azithromycin orally once; if ceftriaxone is not available, we suggest cefixime orally once plus azithromycin orally once, or in case of azithromycin allergy, doxycycline (only for men > 12 years) orally 2 times a day for 7 days (2, ⊕⊕○○).

**R5.6** We suggest for Chlamydia Trachomatis infection: Azithromycin orally once, or Doxycycline (only for men > 12 years) orally 2 times a day for 7 days (2, ⊕⊕○○).

#### Evidence

Complications of epididymo-orchitis are testicular atrophy, with up to 60% of cases demonstrating some degree of atrophy; impaired fertility or sterility; reactive hydrocele [250].

#### Remarks

Considering the possible complications, an appropriate therapy must be set up quickly, and if sequelae develop, adequate follow-up must be set up for the patient, both ultrasound and seminological.

## Testicular tumors

Testicular tumors (TT) in pediatric age (0–18 years) require specific diagnostic and therapeutic management, as they share similarities with adult testicular GCTs but have key differences. It is crucial to distinguish between pre-pubertal and post-pubertal tumors, as biology and treatment protocols have developed differently [268–271]. Pre-pubertal tumors are very rare, generally benign, and rarely associated with distant metastases. In contrast, post-pubertal tumors are more common, malignant in 75% of cases, and often (20–30%) present with distant metastases at onset [272].

Evidence in childhood is limited due to the low incidence of TT. Studies focused on adolescence and the transition age are also lacking, as these age groups are often included in adolescent/young adult (AYA, 15–39 years) studies.

### Epidemiology

Testicular tumors (TT) are the most common solid malignancy in individuals aged 20 to 45 [273], but are less prevalent in pediatric population, although the incidence is increasing in the last decades [274]. Overall, the incidence in pediatric age is approximately 3%. There are two peaks in GCT incidence: one in young children (0–4 years) and another beginning at puberty [275]. TT are rare in childhood (1–2%) [276], but increase to 15% during adolescence [274].

### Pathophysiology

Most testicular tumors in children and adolescents are germ cell tumors (GCTs), with histologic subtypes varying by age. Prepubertal tumors are almost exclusively benign, including mature and immature teratomas and dermoid cysts, while yolk sac tumors are malignant [277]. Post-pubertal tumors are predominantly non-seminomas—choriocarcinoma, yolk sac tumor, embryonal carcinoma, and teratoma, in pure or mixed forms [278], whereas pure seminomas are rare in this age group [279].

Pubertal status is critical for management: post-pubertal teratomas are treated as malignant due to the risk of metastasis, while prepubertal teratomas are always benign. The latest WHO classification also includes certain neuroendocrine variants among prepubertal teratomas [280].

Post-pubertal GCTs, like adult tumors, are often associated with germ cell neoplasia in situ (GCNIS) [2, 281], and signs of testicular dysgenesis, which are absent in prepubertal tumors [282, 283], suggesting distinct developmental origins [284]. Nevertheless, the etiology of pre-pubertal tumors remains unknown.

Extragonadal GCTs can occur in children, typically along the midline (intracranial, pineal, mediastinal) [284]. Sex cord-stromal tumors—including juvenile granulosa cell, Leydig, and Sertoli cell tumors—are rare (10–13%) and usually benign in children, though malignancy is occasionally observed in adolescents and young adults [285–287].

### Clinical picture

Testicular tumors usually present as a firm, palpable mass, often painless but sometimes associated with discomfort or swelling. Detection often occurs via parents, caregivers, or pediatricians. Post-pubertal tumors are often diagnosed at more advanced stages than in children or adults [278, 288, 289]. In adolescents, diagnosis is frequently delayed due to reluctance to report symptoms [290]. and the transition from pediatric to general care [291].

### Diagnostic evaluation

#### Recommendations and suggestions

**R6.1** We recommend performing scrotal ultrasound (US) in the presence of a palpable testicular mass, elevated serum tumor markers (STMs), or retroperitoneal/visceral masses (1, ⊕⊕⊕⊕).

**R6.2** We recommend regular testicular examination and/or testicular US for individuals with risk factors, starting at the onset of puberty (Expert opinion).

**R6.3** We suggest regular testicular US starting at puberty for patients with incidental finding of testicular microlithiasis and other known risk factors for testicular tumors (2, ⊕⊕○○).

**R6.4** We recommend measuring serum tumor markers (α-FP, βhCG, LDH) at diagnosis, prior to any treatment, and during follow-up in post-pubertal patients. In pre-pubertal patients α-FP is sufficient to discriminate between malignant and benign tumors (1, ⊕⊕⊕⊕).

**R6.5** In cases of small lesions with indeterminate findings at testicular US and normal STMs, we suggest repeating the US within 8 weeks. For selected indeterminate cases, consider second-levels exams such as magnetic resonance imaging (MRI) or contrast-enhanced ultrasound (CEUS), (2, ⊕⊕○○).

#### Evidence

Risk factors for GCT are detailed in Table 10. Testicular cancer is etiologically linked to testicular dysgenesis syndrome (TDS), a condition characterized by poor testicular development, dysfunctional testicular somatic cells, and genital malformations such as cryptorchidism [292] and hypospadias (see specific sections). TDS likely results from a combination of inherited genetic variation and environmental factors acting during early development, leading to impaired germ cell differentiation in adulthood [2, 281]. Additional risk factors include birth defects [293], a family history of GCT in a first-degree relative [294] and personal history of GCT [295]. Conflicting data exist in regard with testicular microlithiasis, which is associated with testicular tumor only in patients with additional risk factors [296–299]. A recent metanalysis including 595 children with testicular microlithiasis concluded that microlithiasis was not associated with testicular malignancy during childhood. However, the risk increases during transition age and adulthood follow-up, especially when other risk factors coexist [300].

**Table 10:** Risk factors for testicular germ cell tumors (GCT)

Risk factors for testicular GCT
Cryptorchidism
Hypospadia
Impaired germ cell production
First degree relative with GCT
Personal history of GCT
Down syndrome
Low birth weight
Premature birth
Testicular microlithiasis (if other risk factors)

A testicular physical examination is critical for detecting a mass and assessing its characteristics and should always be performed by primary health care providers in all patients, especially in those with risk factors. Testicular US is the diagnostic gold standard to identify testicular lesions (even the not palpable ones) [301, 302] and should be performed in all boys and adolescent with a palpable testicular mass, elevated serum tumor markers, retroperitoneal or visceral masses or in case of risk factors since puberty [303, 304].

STM assessment contribute to diagnosis, staging, and prognosis in testicular lesions. Common STM for GCT are alpha-fetoprotein (α-FP), human chorionic gonadotropin (β-hCG), and lactate dehydrogenase (LDH). α-FP is crucial in pre-pubertal patients as is secreted by yolk sac tumors, the only malignant form in childhood. Evidence suggests that no male over 6 months of age with a testicular lesion and α-FP < 100 ng/mL has a malignant tumor [305]. Therefore, its measurement is particularly useful for therapeutic decision.

In post-pubertal patients, α-FP, β-hCG, and LDH are crucial markers and should always be tested in cases of testicular lesions or suspected extragonadal GCT. βhCG is significantly elevated in choriocarcinoma, about 15–20% of seminomas, and less frequently in embryonal carcinoma. Nonetheless, a negative result for serum tumor markers does not exclude the diagnosis of GCT [306]. Although LDH is a general inflammation marker, high levels indicate a large tumor burden. However, staging and decision-making should not be based on LDH alone [307].

In cases where testicular ultrasound results are indeterminate and serum tumor markers are normal; it is advisable to repeat imaging within 8 weeks for close monitoring. Additionally, in selected cases, more advanced imaging modalities such as CEUS and MRI can be employed to further refine the diagnosis [308–310].

Differential diagnosis should include orchitis, testicular trauma, adrenal rest tumors, extra-testicular tumors or testicular localization of leukemia or lymphoma [301].

After diagnosis confirmation, total body imaging and post-operative STMs are needed for staging. Staging is not required for benign prepubertal tumors [276]. Pre-pubertal staging is based on Children’s Oncology Group (COG) staging system while post-pubertal staging uses American Joint Commission on Cancer (AJCC) TNMS system and IGCCCG system for metastatic disease [272, 311]. The different staging systems for pre-pubertal and post-pubertal patients complicate comparisons across trials and the development of risk-based treatment strategies.

Imaging of the chest, abdomen, and pelvis using cross-sectional techniques is essential for staging, with computed tomography (CT) being the primary approach. In certain cases, such as when there are neurological symptoms, known chest metastases, or elevated βhCG (> 5000 IU/L) or αFP (> 10,000 ng/ml) levels, MRI of the brain may also be indicated. Staging imaging can be conducted either before or after the primary tumor is surgically removed, depending on clinical context and suspicion of malignancy. At least abdomen MRI is recommended in sex cord-stromal tumors as 10% are malignant with poor prognosis [312].

#### Remarks

Educating patients, especially adolescents, on self-examination is a fundamental step for the early diagnosis of testicular tumors and should be the focus of widespread prevention campaigns. Early diagnosis is strongly associated with a better prognosis for these tumors, particularly in adolescents.

The medical history is crucial for accurately identifying risk factors and guiding the differential diagnosis. Pediatricians should inform patients with risk factors, especially those with present or previous cryptorchidism, as well as their parents/caregivers and the general practitioners responsible for their care post-pediatric discharge, about the importance of regular morpho-functional testicular examinations starting at puberty.

In cases where physical examination suggests a testicular mass, is imperative to perform a testicular US at specialized centers with expertise in pediatric and adolescent testicular pathology. This ensures accurate diagnosis and staging and therefore appropriate therapeutic management.

### Therapeutic management

#### Recommendations and suggestions

**R6.6** We recommend a multidisciplinary management approach for TT in specialized centers with expertise in pediatric and adolescent care, paying particular attention to the transition to ensure long term follow-up in adulthood (1, ⊕⊕⊕⊕).

**R6.7** We recommend determining pubertal status before any treatment (1, ⊕⊕⊕⊕).

**R6.8** We recommend radical inguinal orchiectomy in case of suspicious malignant lesion and normal contralateral testis, in post-pubertal TT (1, ⊕⊕⊕⊕).

**R6.9** We recommend performing testis-sparing surgery with intraoperative frozen-section examination in pre-pubertal tumors if STMs are negative (high suspicion of benign lesion) (1, ⊕⊕⊕O).

**R6.10** We recommend discussing testis-sparing surgery with frozen section examination in patients with a high likelihood of having a benign TT which are suitable for enucleation (1, ⊕⊕⊕○).

**R6.11** We recommend sperm banking in all post-pubertal patients, prior to adjuvant treatment, retroperitoneal lymph node dissection (RPLND). In patients without a normal contralateral testis or known subfertility, cryopreservation should be considered prior to orchiectomy (1, ⊕⊕⊕⊕).

**R6.12** We recommend assessing pubertal stages until completion during follow-up, especially in patients who underwent adjuvant treatments or in patients with low-volume survival testicle, (1, ⊕⊕⊕⊕).

**R6.13** We recommend oncology consultation after orchiectomy for proper treatment/follow-up, preferably in centers with expertise in pediatric and adolescent care (1, ⊕⊕⊕⊕).

**R6.14** We suggest routine contralateral testicular US, even over a long period of time during adulthood (2, ⊕⊕○○).

#### Evidence

##### Pre-pubertal tumors

Partial orchiectomy with intraoperative frozen section has become standard practice due to the high rate of benign lesions and should be performed whenever possible [313]. Orchiectomy should be considered for very large lesions occupying the entire testicle, or in case of α-FP > 100 ng/mL [305]. 80% of patients with yolk sac tumors present at clinical stage I. Approximately 80% of patients with yolk sac tumors present at clinical stage I, with the few metastatic cases usually confined to retroperitoneal involvement. Platinum-based chemotherapy is highly effective for treating the disease and is typically administered before RPLND [276].

##### Post-pubertal tumors

If clinical findings suggest unilateral testicular malignancy, radical orchiectomy is the treatment of choice. Partial orchiectomy may be considered for lesions highly likely to be benign based on first and/or second level exams [301, 308, 309], especially if the tumor is small, the patient has an anatomically or functionally solitary testicle, or in case of bilateral synchronous malignancies. An intraoperative frozen section of the mass with an experienced pathologist is recommended [314, 315]. If there is any concern for GCT, a radical orchiectomy should be performed. If the lesion is confirmed benign, the testicle will be preserved.

All post-pubertal boys should be offered sperm cryopreservation before initiating any adjuvant treatment [316–318], included RPLND [319] or prior to orchiectomy in case of abnormal contralateral testis or known subfertility [320, 321].

Treatment regimens for pediatric GCT have largely been based on clinical trial results from adult men. Whether these results apply equally to children with gonadal or extragonadal GCT remains to be established. To minimize side effects (lung toxicity, ototoxicity, nephrotoxicity) some pediatric protocols used reduced dose of bleomycin from once per week to once per cycle (PEb) [322–324] or carboplatin instead of cisplatin [325, 326]. However, there is some evidence that PEb use may have contributed to poorer outcomes in adolescents [278, 327]. Current ongoing trials will help to better understand the correct protocol for young patients.

About half of post-pubertal testicular GCTs present as clinical stage I. In these cases, active surveillance is the current recommendation [328, 329]. The relapse rate is 20–30%, with excellent survival after salvage therapy [330]. In patients with high-risk pathologic features on the orchiectomy specimen (large size, > 44–50% embryonal carcinoma histology, lymphovascular invasion), adjuvant carboplatin-based chemotherapy can be suggested [331].

Radiotherapy has been used for many years, but current evidence demonstrates a non-inferiority of chemotherapy with less impact on fertility, hypogonadism, and onset of second malignancies [332–334]. RPLND is differently recommended based on guidelines: only after chemotherapy for persistent masses > 1 cm, or in stage II patients who have retroperitoneal-only disease and normal STMs [272].

Adolescent patients with non-seminomatous GCTs had slightly improved survival compared with adults, despite presenting with more advanced disease, achieving excellent survival outcomes [288]. Therefore, follow-up can be long and should take into consideration several issues: (1) Leydig cell dysfunction and hypogonadism may prematurely develop in 10–15% of patients, increasing risk of hypogonadism in adulthood [335]. Monitoring gonadal function from the early stages of pubertal development is necessary in these patients [152]. (2) Adult male survivors who underwent chemotherapy face increased risks of early-onset cardiovascular disease and second malignancies. (3) Literature data reported a 1–3% lifetime risk of contralateral TGCT, even after many years especially if the first tumors occur in young age [295]. Routine testicular US is strongly suggested, even over the canonical oncological follow-up period. (4) An increased prevalence of depression is documented among patients with pediatric TT [336], especially in adolescents, who often need psychological support, due to the difficulty in expressing discomfort or addressing concerns with both family members and doctors [337].

#### Remarks

The assessment of pubertal stage is critical for a correct therapeutic management. TT management is decreed by stage, histology, and risk classification and differs between pre-pubertal and post-pubertal patients. The young age of the patient should not necessarily lead to a less aggressive approach, especially given the higher tendency for metastatic disease in post-pubertal young patients. However, the young age necessitates fertility preservation and a more attentive endocrinological follow-up aimed at achieving complete pubertal development. The psychological repercussions of this challenging chapter in the patients’ lives should not be overlooked.

## Testicular torsion

### Epidemiology

Testicular torsion (TT) exhibits varying incidence rates depending on the age group considered, with a characteristic bimodal distribution. The two peak ages of occurrence are in infancy and early adolescence. TT accounts for 10–25% of acute scrotal events in the pediatric population [257, 338–340]. Its incidence is reported as 1 in 4000 males under 25 years and 1 in 1500 males under 18 years [339, 341, 342].

### Pathophysiology

TT occurs when the testis twists around the spermatic cord, compressing the arterial inflow and causing ischemia and acute scrotal pain [343]. Most affected individuals have no underlying conditions [344]. However, congenital anomalies can increase the risk by allowing excessive testicular mobility. The “bell-clapper” deformity, in which the tunica vaginalis wraps abnormally around the spermatic cord, leaves the testis inadequately attached to the posterior scrotum, facilitating torsion [343, 345].

Other risk factors include larger testicular volume, testicular tumors, horizontal testicular lie, previous cryptorchidism, a long intrascrotal spermatic cord, and less commonly trauma or cold exposure [343, 344, 346].

### Clinical picture

TT typically presents with sudden, unilateral scrotal pain [347, 348], often accompanied by nausea, vomiting, a high-riding and firm testis, absent cremasteric reflex, and erythematous scrotal skin [260, 349, 350].

### Diagnostic evaluation

#### Recommendations and suggestions

**R7.1** We recommend prompt and accurate diagnosis of acute-onset scrotal pain through urological evaluation in the emergency department within 6 h, utilizing the TWIST (Testicular Workup for Ischemia and Suspected Torsion) score (1, ⊕⊕⊕⊕).

**R7.2** We recommend direct surgical exploration for all cases of acute-onset scrotal pain without trauma and a high-risk TWIST score (1, ⊕⊕⊕○).

**R7.3** We recommend the use of Power Doppler Ultrasonography of the scrotum as the initial imaging modality, to be performed by a specialist, in cases of acute-onset scrotal pain without trauma and an intermediate or low-risk TWIST score (1, ⊕⊕⊕○).

**R7.4** We suggest, where available, the use of contrast-enhanced MRI to aid in diagnosis, provided it does not delay the diagnostic process. However, we do NOT recommend it in high-risk cases, which should be promptly referred for surgical exploration (2, ⊕○○○).

#### Evidence

TT is considered a surgical emergency that requires prompt identification and expert management, as it can threaten testicular viability, thus causing irreversible damage and potentially impacting future fertility if not promptly (within 6 h) and appropriately treated [347, 351–353]. TT must be differentiated from other potential causes of acute scrotal pain without trauma, though clinical presentation overlaps should be considered [354]. One such condition is appendageal torsion (involving the appendix testis and appendix epididymis, both remnants of the Mullerian and Wolffian systems, respectively), which is more common than TT in prepubertal boys. Another is epididymitis (see also specific chapter), which typically presents with a more gradual onset of pain [347].

The primary goal during the initial evaluation of acute scrotal pain without trauma is to assess the clinical and physical presentation (as outlined in Table 11) to determine the TWIST (Testicular Workup for Ischemia and Suspected Torsion) score. This clinically validated scoring system for pediatric and transitional-age patients (3 months-18 years) has proven to have high positive predictive values [354, 355]. Cases with a high-risk TWIST score, strongly suggestive of TT, should proceed immediately to urological consultation and surgical exploration. Those with an intermediate or low-risk TWIST score and a questionable diagnosis may proceed to Power Doppler Ultrasonography (US) of the scrotum. Power Doppler has been shown to be more sensitive than color Doppler US in evaluating areas with slow blood flow, such as in the prepubertal testis, with a sensitivity of 63% to 86% and a specificity of 97% to 100% [356–358]. The presence of a twisted spermatic cord, often seen as the “whirlpool sign” (characterized by a sudden change in the course of the spermatic cord, forming a spiral twist at the level of the external inguinal ring or within the scrotal sac), and/or absent blood flow within the affected testicle confirms TT and necessitates immediate surgical exploration [354, 359–361]. Conversely, normal or increased blood flow, suggestive of inflammation or appendageal torsion, would guide appropriate treatment [354].

**Table 11:** TWIST (Testicular Workup for Ischemia and Suspected Torsion) score

Clinical variables	Points
Presence of testicular swelling	2 points
Presence of hard testicle	2 points
Absence of cremasteric reflex	1 point
Presence of high-riding testicle	1 point
Presence of nausea/vomiting	1 point
**Score**	**Risk grading**
6–7 points	High risk
1–5 points	Intermediate risk
0 points	Low risk

While MRI techniques were introduced in 2006, they are not typically employed for acute scrotum evaluation due to their time-consuming and costly nature compared to US. However, dynamic contrast-enhanced MRI, which assesses perfusion and apparent diffusion coefficient (ADC), may help differentiate TT from other acute scrotal conditions. A decrease or lack of perfusion in conjunction with lower mean ADC values has been shown to have a sensitivity of 93% and specificity of 100% for diagnosing TT [362–364].

Hematological parameters may assist in the differential diagnosis between TT and epididymo-orchitis, though large multicenter prospective studies are still needed to validate their clinical utility [357, 365].

#### Remarks

Time is critical in the diagnostic evaluation of acute-onset scrotal pain, and the workup should be completed within 6 h of pain onset, considering any potential delays before medical consultation. No diagnostic test should delay surgical exploration if TT is strongly suspected. While TT is less common than other causes of acute scrotal pain, early intervention is essential to preserve testicular viability.

### Therapeutic management

#### Recommendations and suggestions

**R7.5** We recommend rapid surgical treatment of TT, ideally within 6 h of acute scrotal pain onset, to maximize the chances of testicular tissue survival (1, ⊕⊕⊕⊕).

**R7.6** We suggest performing manual detorsion, following the administration of analgesics and sedation, if surgery is not immediately available or while preparing for surgical exploration (2, ⊕⊕⊕○).

**R7.7** We recommend against manual detorsion as a substitute for surgical exploration (1, ⊕⊕⊕○).

#### Evidence

There is a direct correlation between testicular survival and the duration of testicular torsion (TT), as well as the overall ischemic time. A prompt intervention within 6 h of acute scrotal pain onset is associated with a 97.5% testis salvage rate. However, after 25 to 48 h, only a small percentage of the affected testes will survive, depending on the degree of torsion [366–368].

Manual detorsion may be attempted if surgery is not immediately available or while preparing for surgical exploration. The return of blood flow to the affected testicle can be clinically assessed by pain relief and objectively confirmed with Doppler ultrasound. However, manual detorsion should not delay or replace surgical intervention [366, 369].

A transcrotal surgical approach is typically employed to access the affected testicle and perform detorsion of the spermatic cord [257, 370]. Once detorsion is performed, testicular viability must be assessed. If the testicle is deemed viable, it should be permanently fixed within the scrotum through orchidopexy. If the testicle is nonviable or necrotic, it should be removed [277, 371].

Contralateral orchidopexy should also be performed regardless of the affected testicle’s viability, as some congenital malformations (e.g., “bell-clapper” deformity) are bilateral in up to 80% of patients, increasing the risk of TT in the other testicle [372–374].

#### Remarks

A rapid surgical approach is essential in cases of TT, and patients should be preoperatively counseled about the potential need for orchiectomy if the testicle is found to be necrotic or nonviable [375, 376].
